# Validating models of sensory conflict and perception for motion sickness prediction

**DOI:** 10.1007/s00422-023-00959-8

**Published:** 2023-03-27

**Authors:** Tugrul Irmak, Daan M. Pool, Ksander N. de Winkel, Riender Happee

**Affiliations:** 1 Delft University of Technology Cognitive Robotics Department, Leeghwaterstraat, Delft, The Netherlands; 2 Control and Simulation Department, Delft University of Technology, Leeghwaterstraat, Delft, The Netherlands

**Keywords:** Motion sickness, Perceptual modelling, Sensory integration, Sensory conflict, State estimation

## Abstract

The human motion perception system has long been linked to motion sickness through state estimation conflict terms. However, to date, the extent to which available perception models are able to predict motion sickness, or which of the employed perceptual mechanisms are of most relevance to sickness prediction, has not been studied. In this study, the subjective vertical model, the multi-sensory observer model and the probabilistic particle filter model were all validated for their ability to predict motion perception and sickness, across a large set of motion paradigms of varying complexity from literature. It was found that even though the models provided a good match for the perception paradigms studied, they could not be made to capture the full range of motion sickness observations. The resolution of the gravito-inertial ambiguity has been identified to require further attention, as key model parameters selected to match perception data did not optimally match motion sickness data. Two additional mechanisms that may enable better future predictive models of sickness have, however, been identified. Firstly, active estimation of the magnitude of gravity appears to be instrumental for predicting motion sickness induced by vertical accelerations. Secondly, the model analysis showed that the influence of the semicircular canals on the somatogravic effect may explain the differences in the dynamics observed for motion sickness induced by vertical and horizontal plane accelerations.

## Introduction

Motion sickness is a syndrome whereby aggravating body motions trigger autonomic symptoms such as salivating, dizziness, headaches, panting, hot/cold flushes, stomach awareness, nausea and vomiting (Bertolini and Straumann 2016; Bos et al. 2005). Chronic exposure to sickening motions may lead to the sopite syndrome, which is associated with lethargy, fatigue and drowsiness (Matsangas and Mccauley 2014). These symptoms lead to an increase in subjective workload (Irmak et al. 2021a) and may lead to unwanted task desistance. Therefore, understanding and alleviating motion sickness is particularly relevant for automated vehicles. The exact mechanisms of motion sickness and its time evolution, are however, still poorly understood. Many experimental motion paradigms are, however, known to illicit sickness and sickness is encountered in common modes of transport. Sickness dynamics can be very different from paradigm to paradigm. For instance, humans seem to have a wide band-pass sickness sensitivity to pure lateral acceleration, achieving maximum sickness over a frequency band from 0.03 to 0.3 Hz (Donohew and Griffin 2004). For pure vertical acceleration, the sensitivity is narrower, peaking at a distinct centre frequency of approximately 0.2 Hz (O’Hanlon and McCauley 1974). Moreover, while translational accelerations may lead to sickness in minutes, Earth-vertical yaw rotation (which on its own is not sickening) when coupled with roll leads to vomiting within tens of seconds. Sickness thus exhibits a complex dependency on the frequency, amplitude and direction of motion stimuli.

There are two main theories of motion sickness causation. Riccio and Stoffregen (1991) argue that motion sickness is caused by postural instability (known as postural instability theory of motion sickness). Others argue that sickness occurs due to a mismatch between sensed sensory signals and the sensory signals expected by the brain (Bos 2011) (sensory conflict theory), and that postural instability is a consequence of such mismatch. Overall, we find the theoretical premise put forth by Bos (2011) convincing enough to seek solutions to motion sickness modelling within the sensory conflict framework.

Reason (1978) already argued that sickness was due to sensory conflict. This conflict is the difference between sensed sensory signals and the sensory signals predicted by the brain. The predictions originate from an internal model, which takes the form of a neural store. The neural store can be interpreted as a memory for concurrent patterns of efferent signals (e.g. motor commands) and re-afferent signals (i.e. sensory signals) (Held 1961). Oman (1982) likened this conceptual model to the manner by which a Luenberger observer (LO) operates. The LO uses an internal model of the system (body) and sensor dynamics to estimate the state of the system. Due to the imperfect and noisy nature of the sensory signals, one cannot use the sensor measurements directly. Instead, the true states of the system must be observed (estimated) by using sensory information together with an internal model of the system itself. These estimated states are then used for task planning and execution. To quantify estimation accuracy, the central state estimates are passed through the internal model of sensory dynamics and compared with the actual sensory signals. The resulting error is the estimation error, or the sensory-expectancy conflict. This conflict is used to drive the state estimation towards the true state and to adapt the parameters of the internal model, such that they make better predictions. It is hypothesized that the conflict accumulates over time in a process that resembles leaky integration, resulting in motion sickness.

**Fig. 1:**
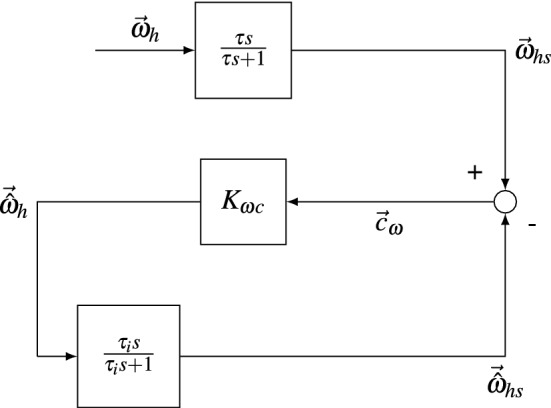
The hypothesized mechanism of velocity storage. The real angular velocity of the head $\vec {\omega }_\textrm{h}$ is measured by the semicircular canals. This measurement is imperfect and results in the high-pass filtering of the head angular velocity into $\vec {\omega }_{\textrm{hs}}$. This is compared with the output of the internally predicted semicircular canal output, $\vec {{\hat{\omega }}}_{\textrm{hs}}$. The difference between the two is the conflict and is passed through the gain $K_{\omega c}$ to give an estimate of the real head angular velocity, $\vec {{\hat{\omega }}}_\textrm{h}$

A more specific example of this general principle was used in Merfeld et al. (1993) to model the phenomenon of velocity storage. When subject to constant velocity earth vertical yaw rotations, the rate of neural spikes originating from the hair cells of the semicircular canals decay toward baseline in 4–5 s. This is because, due to viscous forces, the relative velocity between the endolymph fluid and the hair cells is a transient phenomenon. Velocity storage is the apparent extension in the time constants of angular velocity perception and the angular vestibular-ocular reflex, compared to the raw semicircular canal signal. In Merfeld et al. (1993), it was hypothesized that the velocity storage manifested itself due to the actions of the central nervous system (CNS) similar to the LO shown in Fig. 1.

Here, the semicircular canals sense the head angular velocity $\omega _\textrm{h}$ with first-order high-pass dynamics with a time constant of approximately 4–5 s (Merfeld et al. 1999), resulting in $\omega _{\textrm{hs}}$. The CNS estimates the real head angular velocity. For this, it compares the output of the internal model, which is the expected sensed head angular velocity ${\hat{\omega }}_{\textrm{hs}}$, with the actual sensed head angular velocity $\omega _{\textrm{hs}}$. It uses the conflict between the two to drive the observed angular velocity ${\hat{\omega }}_\textrm{h}$, until the actual sensed and internally estimated velocities match.

In a spatial orientation model (SOM), which Fig. 1 shown above is an example of, the concept of the LO is generally simplified to consider only the sensors and their internal representations, disregarding internal models of the body. The main purpose of these models is to develop an understanding of the systems governing motion perception and reflexive actions in humans. Here, for example, motion perception may be measured by asking for participant reports of their subjective vertical, or their angular velocity by turning a hand dial (Bertolini et al. 2011; Correia Gracio et al. 2013; De Winkel et al. 2018). Reflexive actions may be measured by electro-oculography for eye movements and IMUs for posture. As such, SOMs have seen use in a wide range of contexts: from studying how erroneous motion perception may lead to air traffic accidents (Newman et al. 2012; Borah et al. 1988), to the underlying causes of vestibular disorders and courses of diagnosis and treatment (Holly and Harmon 2013).

The SOM framework (as shown in Fig. 1) naturally results in conflict terms. Although there are qualitative differences between motion perception and reflexive actions, motion sickness per se appears to be mainly related to low-frequency movements, where perception and action are often in agreement (Merfeld et al. 2005a). Moreover, Merfeld et al. (2005b) find that perception is best modelled by the internal model framework. This framework has also been the basis of various motion sickness models. For instance, Zupan et al. (1994) and Denise (1993) extended their perception and action sensory weighting model (SWM) to predict motion sickness experienced during OVAR. Here the conflict was generated between the central state estimate and a *coherence copy* of the head angular velocity, which is the same signal, but computed from other centrally coded variables (for example, the primary organs measuring head angular velocity are the semicircular canals, but head rotation with respect to the gravity vector may also be computed from otolith information). Likewise, Bos and Bles (1998) independently extended their SOM to predict the frequency response of sickness to vertical translational accelerations (VTA). Unlike the SWM, this model hypothesized that the only conflict of importance was the conflict between the sensed vertical and the estimated vertical, and was thus named the subjective vertical model (SVM). Predictions made by this model provided an accurate match to empirical sickness data collected by O’Hanlon and McCauley (1974), and has recently been extended to model the effect of learning exogenous motion dynamics on motion sickness development (Wada 2021).

Although SOMs all have the purpose of enhancing our understanding of the systems governing motion perception and reflexive actions, the proposed models have very different structures. Most models focus on vestibular-only perception, reflecting darkness or eyes-closed conditions or conditions where visual motion perception is less relevant. Likewise, this paper focuses on vestibular-only perception. Here, structural differences between SOMs are mainly related to the various mechanisms employed to perform gravito-inertial ambiguity resolution. This ambiguity resolution is necessary because the otoliths do not report inertial acceleration separately from gravity acceleration. Instead, due to Einstein’s equivalence principle, these are reported in the form of a combined vector named the gravito-inertial acceleration or specific force. For appropriate actuation of effectors, this combined vector must be decomposed into acceleration and gravity.

For instance, the SVM performs gravito-inertial ambiguity resolution by first pre-filtering the input gravito-inertial acceleration. The low-pass filter output is the sensed gravity and the high-pass filter output is the sensed acceleration; this is known as frequency segregation. Other models do not include such an explicit frequency segregation. Such differences lead to different conflict dynamics, and to different sickness predictions.

In the present study, we aim to identify pathways and underlying dynamics that predict motion sickness. To do so, we compare structurally different vestibular-only SOMs, taking into consideration the accumulated body of motion perception and motion sickness data. The work will not be limited to specific conflicts or degrees of freedom, but will explore the relationship between the conflict magnitude of the different sensory channels in various well-known motion paradigms. Ultimately, this study should contribute to models that can serve to prevent motion sickness when used to design technology. This is particularly relevant in the context of self-driving car sickness (Salter et al. 2019; Diels and Bos 2016).

We will compare three structurally distinct SOMs: the subjective vertical model (SVM) (Bles et al. 1998; Bos and Bles 1998, 2002a) where the final model implementation is based on Kamiji et al. (2007), the multi-sensory observer model (MSOM) (Newman 2009) and the particle filter model (PFM) (Laurens and Droulez 2007, 2008). The choice of these models is motivated by a number of considerations. The SVM has been used extensively as the basis of a number of motion sickness studies in ships and cars (Kamiji et al. 2007; Wada et al. 2015; Turan et al. 2009; Khalid et al. 2011; Wada 2021). The MSOM model was developed principally to evaluate disorientation in the context of aircraft operation (Newman et al. 2012; Kravets et al. 2021). The choice of the PFM is aimed at including probabilistic modelling to study motion perception and sickness. The particle filter is an optimal estimator used for highly nonlinear state estimation without the assumption of Gaussian noise. This is contrary to the other two methods. Filtering performance may yield novel insights, particularly for highly nonlinear motions experienced during, for instance, the cross-coupled coriolis motion paradigm. Overall, comparing model performance allows for the identification of responsible mechanisms.

It should be noted that there are other models, such as the previously mentioned SWM. However, the key mechanisms within these models overlap with the chosen three. Inclusion of these models would therefore increase the volume of work, but is unlikely to provide additional insights. Where possible, we linearized the SOMs and performed an analytical evaluation of their conflict dynamics. We then performed simulations of the full model responses to various well-known perception and sickness motion paradigms. In this study, the models were tuned independently to fit both the sickness and the perception data available in the literature. The models, along with the two tunings, were then validated against a second sickness data set using a range of data from the literature (Dai et al. 2003; Howarth and Griffin 2003; Donohew and Griffin 2004; O’Hanlon and McCauley 1974; Bijveld et al. 2008; Cian et al. 2011).

## Models and methods

In this study, we followed a three-step methodology:

1. We linearized the models and performed an analytical analysis of the sensory-expectancy conflict terms. This was done for translational acceleration inputs.
2. Model parameters were tuned to literature data sets for both motion perception and sickness. This allowed us to further quantify the validity of the models and evaluate the relationship between perception and motion sickness.
3. Numerical simulations were then run using the full models, for both the simple motion input cases and the more complex input cases. The simulated sensory conflicts were then fed through a motion sickness accumulation model. The sickness predictions resulting from this were compared with experimental sickness observations from a validation data set.

### Models

We selected three structurally distinct SOMs as a representative sample of the variety of models available in the literature. These models are the SVM, the MSOM, and the PFM. The vestibular inputs to the models are head gravito-inertial force ($\vec {f}_\textrm{h}$) and head angular velocity ($\vec {\omega }_\textrm{h}$). Head gravito-inertial force is measured by the otolith vestibular organ and is a sum of the inertial force and gravity. Head angular velocity is measured by the semicircular canals. The inputs are all in the head frame of reference. The model outputs are estimates of: head referenced gravity ($\vec {{\hat{g}}}_\textrm{h}$), head referenced translational acceleration ($\vec {{\hat{a}}}_\textrm{h}$), head referenced velocity ($\vec {{\hat{v}}}_\textrm{h}$), head referenced angular velocity ($\vec {{\hat{\omega }}}_\textrm{h}$) and an estimate of the orientation ($\vec {{\hat{\theta }}}$) of the head with respect to earth.

It should be noted that all three considered models assume passive motion. This means that the models represent “open-loop” vestibular perception, in the sense that body dynamics and self-motion inputs are not considered. In addition to this, no anticipatory effects (as included in Wada et al. (2015)) or efference copies/internal models that would be active for self-generated motion are accounted for.

The vestibular inputs required for the numerical simulations are computed by modelling a hypothetical motion simulator used to generate the motions (e.g. OVAR) as a kinematic chain. Here, a three-joint system is formulated using standard Denavit–Hartenberg notation and the experienced motions are computed in a feed-forward manner. The motion implementation is documented in detail in Appendix A. All motion paradigms are simulated in darkness, using the vestibular-only models. All simulations were run with a time step of 0.01 s using the Runge–Kutta method for the MSOM and SVM, and the Euler method for the PFM. The computational time requirements were much larger for the PFM, which was simulated with 800 particles. In the following, we describe each model in detail.

#### Subjective vertical model (SVM)

**Fig. 2:**
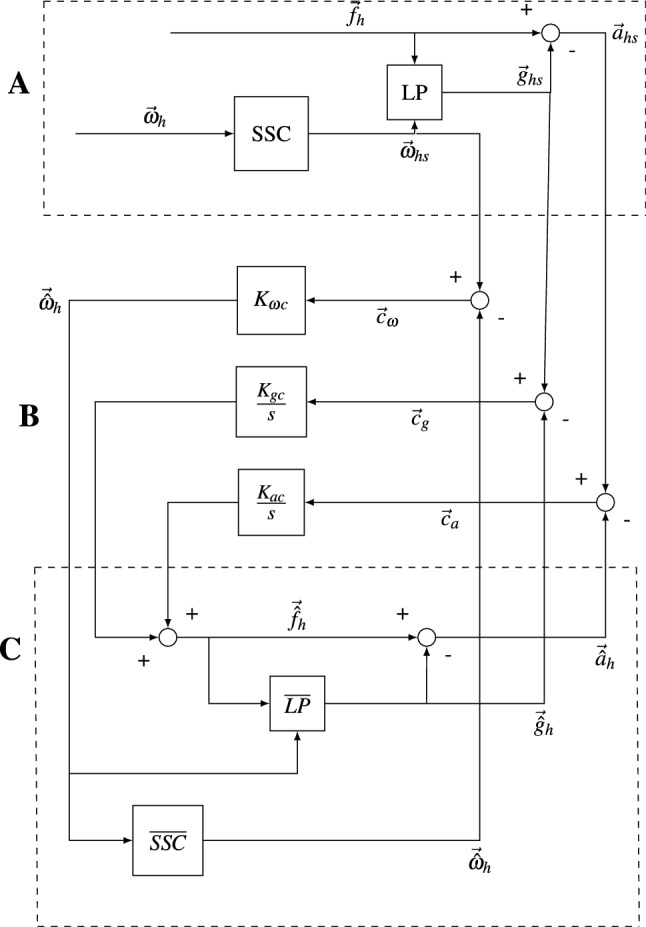
The subjective vertical model. It is parameterized by: $K_{\textrm{ac}}$ is the acceleration feedback gain into the internal model in the form of an integral feedback $\frac{K_{\textrm{ac}}}{s}$, $K_{\textrm{gc}}$ is the gravity feedback gain into the internal model in the form of an integral feedback $\frac{K_{\textrm{ac}}}{s}$, $K_{\omega c}$ is the angular velocity conflict feedback gain into the internal model in the form of a proportional feedback *SSC* and ${\overline{SSC}}$ are first-order high-pass filters representing sensor dynamics of the semicircular canals, *LP* and ${\overline{LP}}$ are first-order low-pass filters capturing gravity estimation according to the Mayne equation

The SVM, shown in Fig. 2, is composed of three main parts: **A** the sensing module, **B** the feedback gain module and **C** the internal model module. The head referenced gravito-inertial force and angular velocity is input through **A** to create sensed gravity, acceleration and angular velocity. To do this it is assumed that the central nervous system first conducts preprocessing in the form of frequency segregation on the gravito-inertial force, by high- and low-pass filtering it into accelerations and gravity components, respectively. The sensed gravity from this operation is then summed with the change in the orientation of the gravity vector as sensed by the semicircular canals. Both operations can be expressed with the so-called Mayne equation (Mayne 1974):

1 $$\begin{aligned} \frac{\textrm{d} \vec {{\hat{g}}}_{\textrm{hs}}}{\textrm{d} t}=\frac{1}{\tau _{\textrm{lp}}}(\vec {f}_\textrm{h}-\vec {{\hat{g}}}_{\textrm{hs}})-\vec {\omega }_{\textrm{hs}} \times \vec {{\hat{g}}}_{\textrm{hs}} \end{aligned}$$

Here, $\vec {{\hat{g}}}_{\textrm{hs}}$ is the sensed estimated gravity vector, $\tau _{\textrm{lp}}$ is the time constant of the low-pass filter and $\vec {\omega }_{\textrm{hs}}$ is the head angular velocity sensed by the semicircular canals.

The sensed states of acceleration, gravity and head angular velocity are then compared to the internal estimates of these states. The difference between the sensed and the internal state estimates is called the *sensory conflict*. The sensory conflict terms are given as: angular velocity conflict $\vec {c}_{\omega }$, gravity conflict $\vec {c}_{\textrm{g}}$ and acceleration conflict $\vec {c}_\textrm{a}$. The conflicts are then fed back into the internal model via the gain module **B**.

In the feedback gain module, the gravity and acceleration conflicts are passed through the integral gains, $\frac{K_{\textrm{gc}}}{s}$ and $\frac{K_{\textrm{ac}}}{s}$, while the angular velocity conflict is passed through a proportional gain, $K_{\omega c}$. These outputs are summed to create the estimated gravito-inertial force. This is passed to the internal model module **C**, which has the same structure as the sensing module **A**. The human perceives and reports the subsequent estimates, $\vec {{\hat{g}}}_\textrm{h}$, $\vec {{\hat{a}}}_\textrm{h}$ and $\vec {{\hat{\omega }}}_\textrm{h}$ during motion perception experiments. The purpose of the internal model **C** is to track the sensed quantities. Bos and Bles (1998) argue that it is the error in the tracking, which is caused by a frequency-dependent phase lag, that leads to sickness.

For translational acceleration inputs, the conflict terms of the model shown in Fig. 2 are derived in Appendix C. This results in the following transfer functions:

2 $$\begin{aligned} \frac{\vec {c}_\textrm{g}(s)}{\vec {f}_\textrm{h}(s)} = \frac{s}{\tau _{\textrm{lp}}s^2 + (1 + K_\textrm{a}\tau _{\textrm{lp}})s + K_\textrm{g}} \end{aligned}$$

3 $$\begin{aligned} \frac{\vec {c}_\textrm{a}(s)}{\vec {f}_\textrm{h}(s)} = \frac{s^2 \tau _{\textrm{lp}}}{\tau _{\textrm{lp}}s^2 + (1 + K_\textrm{a}\tau _{\textrm{lp}})s + K_\textrm{g}} \end{aligned}$$

In Eq. 3 the acceleration conflict $\vec {c}_\textrm{a}$ has high-pass dynamics. As stated in Sect. 1, sickness due to horizontal accelerations has band-pass behaviour. Therefore, $\vec {c}_\textrm{a}$ is inappropriate for sickness modelling. The gravity conflict $\vec {c}_\textrm{g}$ has the desired band-pass dynamics. As seen in Eqs. 2 and 3, the semicircular canal dynamics do not influence the conflicts due to perceived horizontal and vertical accelerations. This also means that the only way to create a difference in the dynamics of the sickness response between vertical and horizontal accelerations is by having a different set of values for the parameters $\tau _{\textrm{lp}}$, $K_\textrm{g}$ and $K_\textrm{a}$ for the different motion directions. Note that due to a lack of a coupling between acceleration input and angular velocity perception, an angular velocity conflict is absent without a rotational input $\omega _\textrm{h}$ in the SVM.

**Fig. 3:**
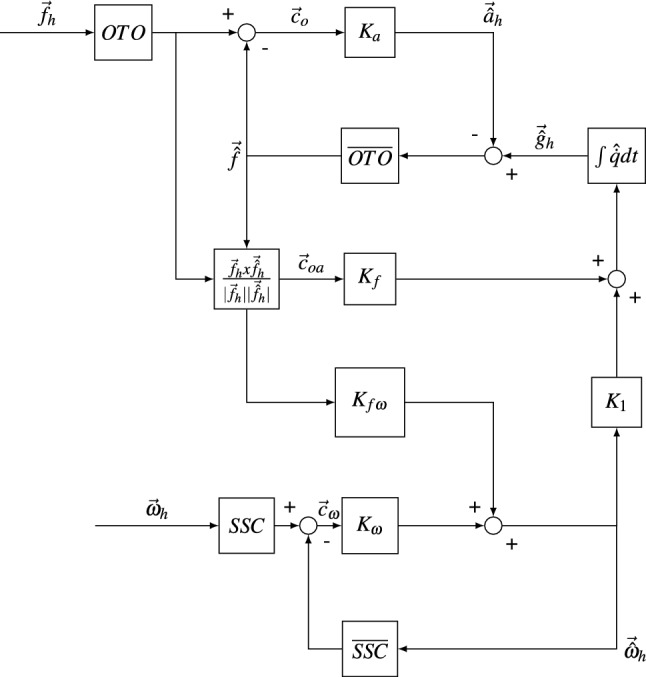
The multi-sensory observer model. It is parameterized by: $K_\textrm{a}$, which multiplies the otolith magnitude conflict to create the internally estimated acceleration $\vec {{\hat{a}}}_\textrm{h}$, $K_f$, which multiplies the otolith angle conflict to create the angular velocity computed from the angular difference between the internally estimated and sensed gravito-inertial force $\vec {{\hat{f}}}_\textrm{h}$ and $\vec {f}_\textrm{h}$, $K_{f\omega }$, which has the same function but instead of being used to compute a new gravity estimate it is summed to create an internal estimate of angular velocity $\vec {{\hat{\omega }}}_\textrm{h}$, and $K_{\omega }$, which multiplies the angular velocity conflict. The subsequent signal is summed with the estimate of angular velocity coming from $K_{f\omega }$. *SSC* and ${\overline{SSC}}$ are first-order high-pass filters denoting the semicircular canals and the internal model of the semicircular canals, respectively, and lastly, *OTO* and ${\overline{OTO}}$ are unit transfer functions denoting the otoliths and the internal model of the otoliths, respectively

#### Multi-sensory observer model

The MSOM (Fig. 3) functions as a classical observer model, whereby the sensory afferent signals are directly used by the internal model, which contains an internal representation of sensor dynamics coupled to the physical relationships between different states. It has a more integrated structure than the SVM and cannot be easily segmented into different modules. As in the SVM, the inputs to the model are the gravito-inertial force $\vec {f}_\textrm{h}$ and the head angular velocity $\vec {\omega }_\textrm{h}$, both in the head frame of reference. These sensory signals are compared to an internally expected gravito-inertial force $\vec {{\hat{f}}}_\textrm{h}$ and angular velocity $\vec {{\hat{\omega }}}_\textrm{h}$. These lead to the otolith magnitude and angular velocity sensory conflict terms, $\vec {c}_\textrm{o}$ and $\vec {c}_{\omega }$. The angular velocity conflict is multiplied by a gain $K_{\omega }$ and then added to the angular velocity calculated from the otoliths to create the estimated head angular velocity $\vec {\hat{\omega _\textrm{h}}}$. This angular velocity is multiplied by a gain $K_{1}$ (where $K_1 = \frac{K_{\omega f} +1}{K_{\omega }}$) and added again to the head angular velocity estimated from the otoliths. This is then used to rotate the gravity vector toward the internally expected angular position of the head. In this, as it has implications for model dynamics, it is important to note (as discussed below) that the magnitude of the estimated gravity $\vec {{\hat{g}}}_\textrm{h}$ is always $-\,9.81$ ms$^{-2}$. Only the vector components of this estimation change when rotated by angular signals from the otolith and the semicircular canal path. The newly computed gravity is summed with the acceleration estimate to create the internally expected gravito-inertial force $\vec {{\hat{f}}}_\textrm{h}$. The angular signal from the otoliths is derived by computing the angular difference between the expected and the sensed gravito-inertial force. This is the third sensory conflict, called otolith angle conflict, $\vec {c}_{\textrm{oa}}$. This is obtained by taking the cross-product of $\vec {f}_\textrm{h}$ and $\vec {{\hat{f}}}_\textrm{h}$.

**Fig. 4:**
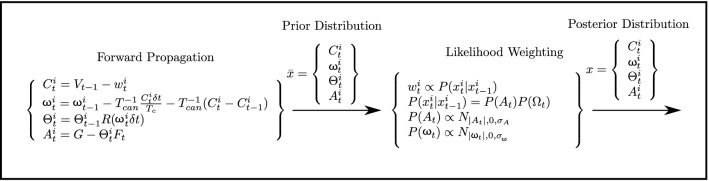
The particle filter model. It is parameterized by: the weighting factor $w^i_t$ given by a multiplication of Gaussian priors (of mean zero and variance $\sigma _A$ and $\sigma _{\omega }$) on the inertial acceleration $P(\vec {A}_t)$ and angular velocity $P(\omega _t)$, the head-to-canal transformation matrix $T^{-1}_{\textrm{can}}$, the integration time step $\delta t$ and the canal time constant $T_\textrm{c}$

In the MSOM, the estimated gravity vector only changes due to a rotation signal from the otolith angle conflict or the semicircular canals. Unlike the SVM, the magnitude of the estimated gravity ${\hat{g}}_\textrm{h}$ is always assumed to be constant at $-\,9.81$ ms$^{-2}$. This causes important differences in the dynamics of the MSOM compared to the dynamics of the SVM. For instance, for vertical acceleration inputs there is no rotation signal, so the estimated gravity remains constant and the remaining gravito-inertial force is attributed to acceleration. Therefore, the only conflict that occurs during vertical acceleration is the otolith magnitude conflict, for which the linearized frequency response takes the form:

4 $$\begin{aligned} \frac{c_{\textrm{oz}}(j\omega )}{f_{\textrm{hz}}(j\omega )} = \frac{1}{1 - K_\textrm{a}} \end{aligned}$$

Irrespective of the value of $K_\textrm{a}$, equation 4 shows that the conflict response will be independent of acceleration frequency. As stated in Sect. 1 vertical sickness sensitivity in humans is narrow band-pass, with a centre frequency of approximately 0.2 Hz. Therefore, this is inappropriate for sickness prediction. One can change the gain $K_\textrm{a}$ to integral or differential, which will result in high-pass or low-pass dynamics, respectively, but this would not resolve the fundamental issue. Even though more complex feedback gain combinations may be employed to correct this, any changes in the conflict dynamics would also change the dynamics of the acceleration estimate from high-pass to a more complex frequency response, due to

5 $$\begin{aligned} {\hat{a}}_{\textrm{hz}}(j\omega ) = K_\textrm{a} c_{\textrm{oz}}(j\omega )\ . \end{aligned}$$

Experiments thus far have not, however, shown such complex acceleration perception responses (Merfeld et al. 2005a). For lateral acceleration inputs, if one assumes no cross-talk between otolith computed angle and the integrated semicircular canal output by setting $K_{f\omega }$ to zero (as derived in Appendix D), the frequency response of the conflict terms take the form:

6 $$\begin{aligned}{} & {} \frac{c_{\textrm{oy}}(s)}{f_{\textrm{hy}}(s)} = \frac{s}{s(1-K_\textrm{a}) + K_f} \end{aligned}$$

7 $$\begin{aligned}{} & {} \frac{c_{\textrm{oay}}(s)}{f_{\textrm{hy}}(s)} = -\frac{c_{\textrm{oy}}}{|g|} \end{aligned}$$

In Eq. 6 it can be seen that indeed the otolith magnitude error response is high-pass. Likewise, the otolith angle error is just a factor of $g_z$ smaller in magnitude. Just as for vertical inputs, the gains can be set in such a way that the resulting conflict frequency response approximates the observed lateral sickness response. However, doing so will also affect the high-pass dynamics of the acceleration estimate.

#### Particle filter model

As shown in Fig. 4, the PFM works on a distinctly different principle than the SVM and MSOM. It is composed of two major parts: the forward propagation, which consists of the prior distribution and the likelihood weighting and the subsequent re-sampling, which determines the final posterior distribution. The posterior distribution is propagated solely from the semicircular canal signal. Using the notation used by Laurens and Droulez (2007), for any given particle, *i* Gaussian noise $w^{i}_t$ is added to the average sensed canal signal (at the previous time step) $\vec {V}_{t-1}$ creating $\vec {C}^{i}_t$. From this the angular velocity of the particle, $\Omega ^{i}_t$ is computed by directly inverting the canal equation:

8 $$\begin{aligned} \omega ^{i}_t = \omega ^{i}_{t-1} - T^{-1}_{\textrm{can}}\frac{\vec {C}^{i}_t\delta t}{T_\textrm{c}} - T^{-1}_{\textrm{can}}\left( \vec {C}^{i}_t - \vec {C}^{i}_{t-1}\right) \end{aligned}$$

This angular velocity integrated over time gives the rotation of the particle *i* from time $t-1$ to *t*: $R(\Omega ^{i}_t \delta t)$. This, when multiplied with particle orientation from $t=0$ to $t -1$ gives the orientation of each particle at *t* specified by $\Theta ^{i}_t$. This is the particle-to-earth transformation matrix, from which the earth referenced acceleration $\vec {A}^{i}_t$ can be computed. Note that the magnitude of gravity |*G*| (Fig. 4) is earth referenced and assumed, just like in the MSOM, to be constant at $-\,9.81$ ms$^{-2}$. Collecting all the states gives the prior distribution. Each particle is then weighted with respect to the state transition probabilities. The assumption is that the brain assigns the greatest probability to stationary states. Thus, the particles that are closest to zero acceleration and angular velocity are weighted the highest. This weighing factor is given by $P(\vec {A}_t)P(\Omega _t)$, which is defined to have a Gaussian distribution. Thus, the further down the tails of the distribution the particle acceleration or angular velocity is, the lower its weight. After the weighing, the particles are sampled with a probability proportional to their weight.

For the PFM, no conflict terms are explicitly present, but an angular velocity conflict can be calculated in the form

9 $$\begin{aligned} \vec {c}_{\omega }(t) = SSC(\vec {\omega }_\textrm{h}(t)) - {\overline{SSC}}(\vec {{\hat{\omega }}}_\textrm{h}(t))\ \end{aligned}$$

Here *SSC* is the function giving the output of the semicircular canals, $\vec {\omega }_\textrm{h}$ is the angular velocity of the head and $\vec {{\hat{\omega }}}_\textrm{h}$ is the internally estimated angular velocity of the head. There are, however, no otolith or acceleration/gravity conflict terms. This is because inverting the otolith equation to estimate acceleration and gravity requires the sensed gravito-inertial force. Summing the gravity estimate and acceleration estimate gives back the same sensed gravito-inertial force, meaning an otolith conflict cannot be calculated for the PFM.

### Motion sickness accumulation model

An example of how the conflict terms are mapped to the accumulation of a sickness metric is given for the SVM (Bos and Bles 1998). Here, the gravity conflict, $\vec {c}_\textrm{g}$ is taken, and its magnitude is computed and scaled according to a Hill function of the form

10 $$\begin{aligned} h = \frac{(|\vec {c}_\textrm{g}| /b)^n}{1 + (|\vec {c}_\textrm{g}| /b)^n}\ \end{aligned}$$

where *h* is the scaled conflict and *n* and *b* are the parameters of the chosen Hill function. After this scaling, a second-order system of the form

11 $$\begin{aligned} \textrm{MSI} = \frac{P}{(\mu s +1 )^2}h\ \end{aligned}$$

integrates the conflict over time to predict motion sickness incidence (MSI), which is the percentage of people that have vomited from time zero to time current. Where *P* and $\mu$ are the parameters dictating how the conflict is accumulated. Thus, the SVM includes four additional parameters (*b*, *n*, *P* and $\mu$) to describe motion sickness accumulation.

Khalid et al. (2011) tried to extend the SVM to describe horizontal motion sickness. Here they note that the subjective horizontal conflict is like a high-pass filter ”*which should be adjusted (by low-pass filtering) before being translated into the MSI*”. However, with such a filter, any experimentally observed frequency dependency of motion sickness susceptibility could be matched, reducing model uniqueness. This would also mean the need for at least two extra parameters to obtain band-pass characteristics, and these would not have any physiological basis.

Therefore, in the current study, the absolute value of each conflict term is computed. The consequent terms are then scaled and integrated over time. These two operations do influence the predicted sickness frequency response. However, they are applied to the absolute value of the conflict signal, which has most of its power in its DC bias term (0 Hz), as shown in Appendix B. Such operations therefore have a negligible effect on the frequency response characteristics within the range of frequencies where sickness is observed.

Because the rest of our analysis will evaluate multiple motion paradigms, it is of greater utility to use a simpler system that does not have the free parameters used in Bos and Bles (1998) for sickness accumulation. A simple integrator on the absolute conflict is sufficient. Each conflict term likely has a different importance weighting $W_i$ for motion sickness. This weighting is found by optimizing the sickness predictions compared to measured sickness data. This means the sickness proxy, MS, used for this study is defined as

12 $$\begin{aligned} \textrm{MS} = \int ^t_0 \vec {W} \cdot |\vec {c}(t)|\textrm{d}t\ , \end{aligned}$$

This MS is different but correlated to MSI, which was explicitly fitted to vomiting. Here, $|\vec {c}(t)|$ is a vector whose elements are the magnitude of individual conflict types (i.e. angular velocity conflict and gravity conflict). For a given conflict type this magnitude is computed by assuming unit weight for all the components of this vector, i.e. the first element of $|\vec {c}(t)|$ is $\sqrt{c_{gx}^2 + c_{gy}^2 + c_{gz}^2}$.

Lastly, the current study evaluates all available internal conflict terms and identifies all those relevant for predicting sickness. For the SVM these are the gravity conflict $\vec {c}_\textrm{g}$, acceleration conflict $\vec {c}_\textrm{a}$ and angular velocity conflict $\vec {c}_{\omega }$. For the MSOM these are the magnitude of the conflict between the otolith signal and predicted otolith signal $\vec {c}_\textrm{o}$, angular velocity conflict $\vec {c}_{\omega }$, and otolith signal orientation and predicted signal orientation $\vec {c}_{\textrm{oa}}$. For the PFM, this is the angular velocity conflict $\vec {c}_{\omega }(t)$.

### Model tuning

In this study, the time-domain perception predictions of the models are first given for the parameter values reported in the literature where these, so-called untuned parameters are based on Bos and Bles (2002b), Bos and Bles (1998) and Kamiji et al. (2007) for the SVM, Newman (2009) for the MSOM and Laurens and Droulez (2007), Laurens and Droulez (2008) for the PFM.

We then tuned the models to a number of data sets on empirical observations on motion perception and on motion sickness. Under the SOM framework, there is a coupling of perception and motion sickness dynamics. All models in the literature were tuned to different experimental data sets. Therefore, a *perception tuning* is important for comparing structural, rather than parameter dependent differences between the models. Upon analysing the sickness predictions made from the perception-tuned parameters, it was clear that the perception tuning did not predict sickness accurately. The additional *sickness tuning* allows for the evaluation of how closely the models can fit experimentally observed sickness data, independent of perception. Evaluating both perception-tuned and sickness-tuned results together helps identify whether the SOM framework is effective for explaining motion sickness. Indeed, the fact that this is needed is already indicative of the fact that from a broad standpoint, the models don’t accurately describe the link between perception and sickness.

#### Perception tuning

Models were fitted to perception data for centrifugation, earth vertical axis rotation (EVAR) and off vertical axis rotation (OVAR). Data for centrifugation were obtained for eccentric rotations at $36^\circ \,\textrm{s}^{-1}$ and $60^\circ \,\textrm{s}^{-1}$, associated with centripetal accelerations of 2.41 and 6.7 ms $^{-2}$, respectively (Graybiel and Brown 1951); $200^\circ \,\textrm{s}^{-1}/12.2$ ms $^{-2}$ (Curthoys 1996); and $250^\circ \,\textrm{s}^{-1}/10.3$ ms$^{-2}$ (Merfeld et al. 2000). In these studies, perceived orientations were measured by asking participants to report perceived roll by adjusting a bar to be in line with either the subjective horizontal or the subjective vertical. The angular displacement of the bar is a measure of the perceived tilt. Data for EVAR and OVAR were obtained from Vingerhoets et al. (2005). Seated participants were tilted to $\{0, 15, 30\}^\circ$ relative to Earth-vertical. In the $0^\circ$ and $30^\circ$ tilt conditions, participants were rotated at $30^\circ \,\textrm{s}^{-1}$; in the $15^\circ$ tilt condition, participants were rotated at $\{20, 30, 40, 50\}^\circ \,\textrm{s}^{-1}$. Participants were presented with a laser-projected dot moving across a screen. Using a toggle switch, subjects indicated how the speed of the dot had to be adjusted (faster or slower) in order for it to be perceived as space fixed. Hence, the adjustment of the dot speed was a measure of the perceived rotation velocity.

These conditions were simulated by feeding the corresponding motion signals to the perception models. Centrifugation was simulated for a participant sitting Earth vertically upright at a radius of 6.1 m, 1 m and 0.54 m from the centre of rotation. EVAR was simulated by rotating an earth vertically upright participant about the Earth vertical axis. OVAR was simulated tilting the participant off-vertical such that the long axis of the body makes an angle with the Earth vertical and rotating the participant about the long body axis. To ensure sufficient convergence, all simulations were run for 200 s of simulated time.

The perception models were fitted to the data by minimizing the symmetric mean absolute error (SMAE) between model and empirical data for time-domain signals of rotation perception during EVAR, roll perception during centrifugation, as well as translation and rotation perception during OVAR. In the process of optimizing the model parameters to fit the perception data, the models were first tuned to match an EVAR perception decay time constant of 18.7 s, as reported by Vingerhoets et al. (2005). This time constant reflects the ’velocity storage’ mechanism, which is a neural mechanism that extends the perception of rotational velocity to persist for a period of time even when the semicircular canals have assumed a zero output during constant velocity rotation. This time constant is thought to be of particular importance to motion sickness because it correlates strongly with individual motion sickness sensitivity (Dai et al. 2003, 2007; Young et al. 2003).

The EVAR paradigm was used for perception tuning because it is a pure rotation, which allows a direct estimation of this time constant. The consequence of fixing the EVAR time constant is that it reduces the dimensionality of the final optimization and so allows faster and better convergence. After fitting the models to match this time constant, the other internal model parameters were estimated.

The considered models are overparametrized, which causes many local minima in parameter estimation. Therefore, simulated annealing (SA) was used to estimate the global minimum. SA is a probabilistic technique used to approximate the solution to global optimization problems (Henderson et al. 2006) such as the one encountered here. Ten SA runs of 100 iterations were performed about the literature referenced parameter values. Model parameters belonging to the 10th percentile for lowest prediction error were identified. This resulted in up to 30 candidate parameter sets.

Subsequently, k-means clustering was used to cluster the candidate points to three regions. K-means was deemed appropriate, as parameter sets were grouped closely to each other. The validity of the identified regions was then verified by inspection. Local optimization using the interior-point algorithm was run on the three centroids. This tuning ensured that the final parameters result in representative modelling of perceptual phenomena across a wide domain.

#### Sickness tuning

For the sickness tuning, the perception-tuned models were taken as the initial starting point. The models were then further tuned to available motion sickness data from literature. We used experimentally obtained frequency sensitivities for lateral translational accelerations (LTA) (Donohew and Griffin 2004) and VTA (O’Hanlon and McCauley 1974). These data sets are the only sickness paradigms with reliable data that could be used for optimization. Fitting on frequency sensitivities to sickness is common in motion sickness literature and has been done before for VTA and LTA by Bos and Bles (1998) and Khalid et al. (2011), respectively, to tune the SVM.

### Model validation for sickness

To judge the generalizability of the models, it is important for the validation data set to be independent of the fitting data sets. For this purpose the three models were validated with respect to the predicted and experimentally observed motion sickness magnitude across five frequently used motion paradigms in motion sickness: cross-coupled coriolis perturbation (CCCP) (Dai et al. 2003), pure roll perturbation (PRP) (Howarth and Griffin 2003), LTA (Donohew and Griffin 2004), VTA (O’Hanlon and McCauley 1974), and OVAR (Bijveld et al. 2008; Cian et al. 2011). Here, CCCP is performed by rotating participants earth vertically whilst rolling them in discrete steps about the naso-occipital axis. For CCCP, the waiting duration between each head roll was 10 s and each head roll took 2 s to perform. For PRP, LTA and VTA, the perturbation frequency was 0.2 Hz.

Unfortunately, there are no experiments that investigate motion sickness for all these paradigms over the same duration and using the same sickness rating scale. Therefore, the relative differences in sickness magnitude between these paradigms had to be inferred. Fortunately, however, the associated experiments have all based their rating system on motion sickness symptoms. Still, the termination points of these experiments are not consistent. For instance, experiments on PRP may not provoke nausea at all, or the number of participants that experience it may be too small for a reliable sickness proxy. For this reason, a commonly shared point of “mild symptoms with no nausea” is taken for this model comparison. The mean duration to reach this rating is used to characterize sickness intensity. Experimental studies on motion sickness led by Griffin (Howarth and Griffin 2003; Donohew and Griffin 2004) report a mean duration of 15 minutes for LTA and greater than 30 minutes for PRP. So, for the motion stimuli used by Griffin, LTA should be at least twice as provocative as PRP. The relative magnitude of VTA compared to LTA is a contentious topic. For example, unpublished observations by Mills and Griffin referred to in Donohew and Griffin (2004) report similar sickness ratings for both VTA and LTA. Golding et al. (1995), however, maintains that the sickness response is actually coupled to posture. Horizontal perturbations, when the participant is earth horizontal (lying down), are about half as provocative as vertical perturbations for an earth vertical participant (upright). On the other hand, horizontal perturbations for an upright participant are 2.5 to 1.8 times as provocative as upright vertical perturbations (Golding et al. 1995). Current models do not account for this possible posture dependency. Hence, in the present study, the tuned models were evaluated for upright seating. Additionally, we assume no rotation of the head for LTA. This is because the rotation expected at the maximum acceleration of 0.89 ms $^{-2}$ at 0.2 Hz is negligibly small (Paddan and Griffin 1988). OVAR experiments in the dark (Bijveld et al. 2008; Cian et al. 2011) show that the duration for mean sickness to reach mild symptoms with no nausea is 2.1 minutes. This is a factor of 7 smaller than that reported for LTA. Lastly, for CCCP, there are no experiments which map the slow rise in sickness over time. This is mostly because sickness induced by CCCP is intense and quick (Dai et al. 2003). Investigators mainly focus on the number of head movements tolerated. In most cases, participants reach mild symptoms after 10-20 head turns. Therefore, the mean duration to reach mild symptoms with nausea can be said to be in the order of tens of seconds and so CCCP is estimated to be 100 times as sickening as the LTA paradigm used by Donohew and Griffin (2004). To summarize, the inferred relative sickness magnitudes are: CCCP 1, OVAR 0.07, LTA 0.01, VTA 0.005-0.02, PRP $< 0.005$.

For validation in the frequency domain, the motion paradigms of LTA from Donohew and Griffin (2004) and VTA from ISO 2631 (1997) were used, because these are the only sickness paradigms with reliable frequency-domain data that provides adequate information to perform an optimization. The frequency-domain data for Donohew and Griffin (2004) is based on the proportion of participants developing mild nausea when subject to horizontal accelerations for up to 30 minutes, whereas for ISO 2631 (1997) it is based on the proportion of participants vomiting when subject to vertical accelerations for up to 2 h. In the experiments from which the data were obtained, the participants’ heads were not constrained, but as head motion measurements are not available, seat motion was used as input to the perception models.

## Model tuning results

Following the procedure outlined in the Model Tuning section, the three models were tuned to data from perception and sickness experiments. Tuning reduces the parameter-dependent variability between the models, highlights the consequences of the structural differences identified in the previous sections, and allows us to evaluate whether perception predictions are consistent with sickness predictions and vice versa.

### Fit to empirical perception time-domain data

**Fig. 5:**
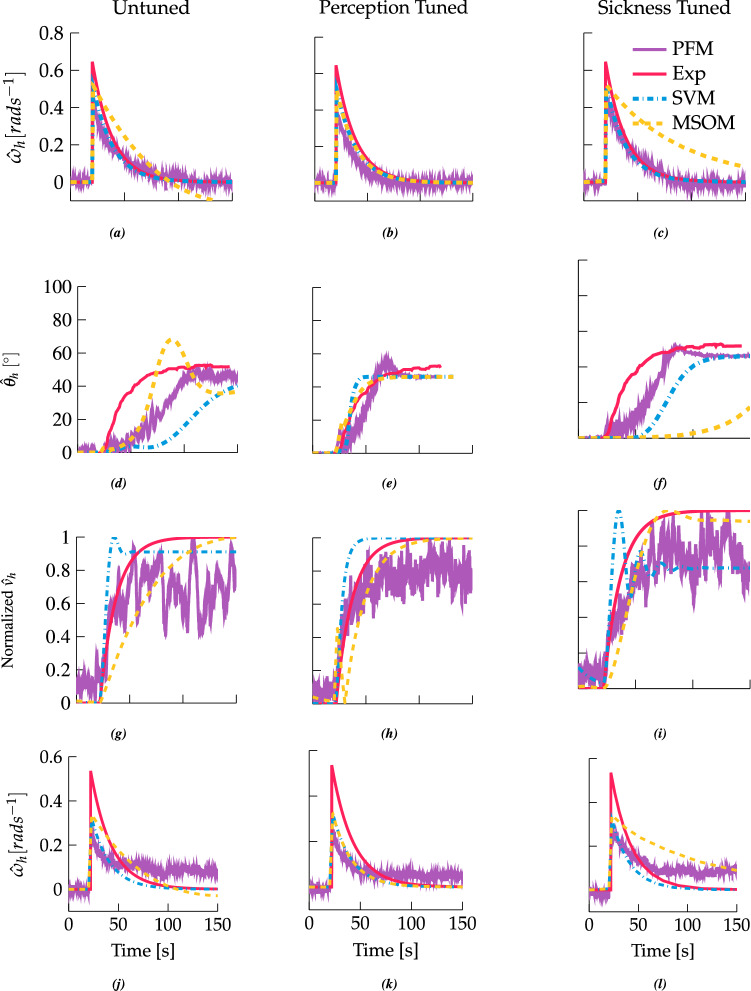
Perception predictions for EVAR, centrifugation and OVAR, according to the PFM (purple lines), SVM (dashed blue) and MSOM (dashed yellow), plotted against experimental data (red lines). The first row shows yaw velocity perception for EVAR. The second row shows roll perception during centrifugation. In the third and fourth rows, the normalized translation velocity perception (obtained by integrating translational acceleration) and rotation perception are shown for OVAR, respectively. The left column shows fits based on parameters obtained from the literature; the centre column perception-tuned fits; the right column sickness-tuned fits. All motion paradigms are performed in darkness

In the following, we evaluate how well models tuned to either perception time-domain data or sickness frequency-sensitivity data predict empirical time-domain perception data. The left column of Fig. 5 shows how the models perform using parameters reported in the literature; the centre and right columns, respectively, show the tuning to aggregated perception and sickness data sets obtained in the present study.

For EVAR (Fig. 5a), the PFM and the SVM behave similarly: both converge exponentially to zero. In agreement with experimental data, the perceived rotational velocity fades in steady-state motion. The MSOM, however, eventually undershoots, indicating perception of counter-rotation to the initial direction of motion. The undershoot here owes to the second-order nature of the semicircular canal dynamics employed by the original paper (Newman 2009). For the perception tuning, the second-order semicircular dynamics used in Newman (2009) are simplified to a first-order system. After tuning, the EVAR time constant is set to 18.7 s for all the perception-tuned models (as shown in Fig. 5b). The lower gain of the PFM for EVAR is due to the canal noise required for the model to function (Fig. 5b). When the models are tuned to sickness (right column in Fig. 5) it is seen that the time constant for the MSOM increases to substantially above experimental observations. The time constant for the SVM is not adapted, because the velocity storage in the SVM plays no role in sickness development due to lateral or vertical translational accelerations.

The second row of Fig. 5 shows roll perception for centrifugation in the dark. The red line indicates the experimental observations by Merfeld et al. (2000). From fitting an exponential model of the form $\Theta _x = A(1-e^{-bt})$ to the perceived roll, the time constant *b* is 26.3 s. Oscillations are observed at the beginning of the simulation for the PFM in Fig. 5d from t = 20 s until t = 80 s. These oscillations are due to the time step used. A large time step means that the estimate of orientation is different from the actual orientation, causing a misestimation of acceleration when the otolith equation is inverted. This phenomenon is proportional to the angular velocity at the point in time and the noise about this point in time. Therefore, under certain motion conditions, the particle filter is under damped. The MSOM was initially tuned to the data from Graybiel and Brown (1951), for which the authors used a lower yaw rate of 60 $^{\circ }\,\textrm{s}^{-1}$ than Merfeld’s yaw rate of 250 $^{\circ }\,\textrm{s}^{-1}$. This higher yaw velocity perturbs the system such that the response overshoots and the estimation requires a longer time to converge. After perception tuning (Fig. 5e), the predicted roll percept by the MSOM no longer has this overshoot. Likewise, the SVM also responds much faster to the stimuli. Lastly, there is a decrease in the time constant of the PFM from 52 to 27 s, which is closer to the experimentally observed value of 22 s (Merfeld et al. 2000). It can be seen that tuning these results to sickness (Fig. 5f) creates responses that do not match the perceptual data. The MSOM now has a very large time constant meaning slow convergence; likewise, SVM is slower also to converge than before.

The final modelled perceptual phenomenon is OVAR. The participant in this case first has a sense of rotation, which transitions into a sense of translation around a conic or cylindrical trajectory. Vingerhoets et al. (2005) evaluated motion perception for two components, rotation and translation. The untuned results for the two components are shown in Figs. 5g, j. In Fig. 5g, the MSOM and the PFM show a steady-state rotation bias. This rotation bias should create the perception of changing heading, which in experiments is only rarely observed (Wood et al. 2007; Vingerhoets et al. 2005). With the perception tuning, as shown on Fig. 5k, this bias is removed for the MSOM. However, removing the bias is not feasible for the PFM without compromising its centrifugation fits. For all models, the sickness tuning (Fig. 5l, i) yields a poorer match with the measured perception results for OVAR.

**Table 1:** SVM parameters untuned, perception-tuned and sickness-tuned; untuned parameters are based on Bos and Bles (2002b), Bos and Bles (1998) and Kamiji et al. (2007)

	SVM				MSOM				PFM		
	Untuned	Perception	Sickness		Untuned	Perception	Sickness		Untuned	Perception	Sickness
$\tau _{ssc}$ [s]	5.7	5.7	5.7	$\tau _{ssc}$ [s]	5.7	5.7	5.7	$\tau _{ssc}$ [s]	4	5.7	5.7
$\tau _{lp_{xy}}$ [s]	5	1.23	15.0	$K_\textrm{a}$	− 4	− 3.2	− 7.2	$\sigma _\textrm{c}$ [$^{\circ }\textrm{s}^{-1}$]	10	10	10
$\tau _{lp_{z}}$ [s]	5	1.23	5.13	$K_f$	4	15.4	0.004	$\sigma _\textrm{a}$ [$\textrm{ms}^{-2}$]	5	0.5	1.5
$K_{ac_{xy}}$	1	0.005	1.2	$K_{f \omega }$	8	0	8.4	$\sigma _{\Omega }$ [$^{\circ }\textrm{s}^{-1}$]	30	26	26
$K_{ac_{z}}$	1	0.005	0.78	$K_{\omega }$	8	2.28	11.2				
$K_{gc_{xy}}$	5	1.88	3.26	$K_{\omega f}$	1	1	1				
$K_{gc_{z}}$	5	1.88	5.88								
$K_{\omega c}$	2.28	2.28	2.28								

MSOM parameters; untuned parameters are published in Newman (2009). PFM parameters; untuned parameters published in Laurens and Droulez (2007) and Laurens and Droulez (2008)

**Table 2:** Symmetric mean absolute error between the predicted and experimental perception results shown for the untuned, perception-tuned and sickness-tuned models

Models	Motion paradigm			
	EVAR	OVAR Trans	OVAR Rot	Centrifugation
MSOM Untuned	0.634	0.215	0.471	0.282
MSOM Perception	0.088	0.210	0.324	0.069
MSOM Sickness	0.699	0.243	0.616	0.640
SVM Untuned	0.088	0.260	0.324	0.297
SVM Perception	0.088	0.237	0.324	0.094
SVM Sickness	0.088	0.333	0.324	0.474
PFM Untuned	0.615	0.303	0.441	0.320
PFM Perception	0.559	0.257	0.564	0.106
PFM Sickness	0.564	0.278	0.552	0.211

The perception and sickness-tuned, as well as the original untuned, parameter values are listed in Table 1. Table 2 shows the overall goodness of fit for all tunings. Here, the perception tuning procedure is overall successful in reducing the error between the perception data set and the model predictions. The most accurate overall perception results are provided by the MSOM, whereas for centrifugation the SVM is of greater accuracy. Sickness-tuned parameters performed poorer in perception than both perception-tuned and untuned, indicating that the models cannot account accurately for both perception and sickness at the same time.

### Perception frequency responses

**Fig. 6:**
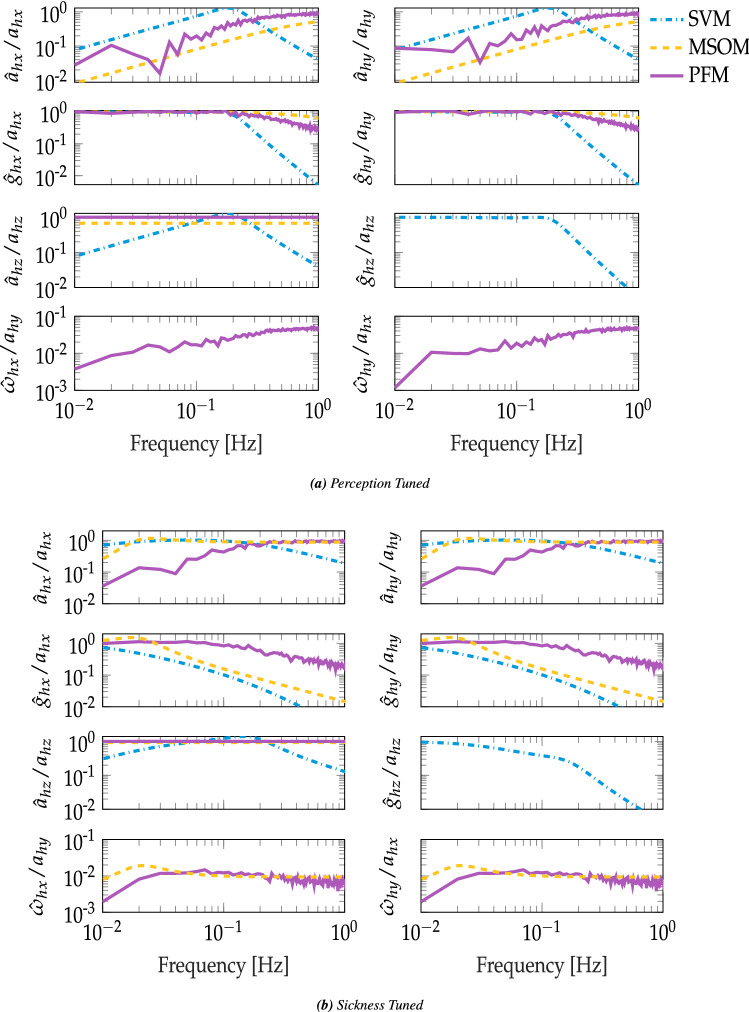
Frequency-domain model responses of perceived motion. **a** responses of the perception-tuned model state estimates in response to small acceleration input whilst earth vertically orientated in darkness. The three lines represent SVM (blue), MSOM (yellow) and PFM (magenta). Input head acceleration is $a_{h}$, estimated head acceleration is ${\hat{a}}_\textrm{h}$, estimated gravity is ${\hat{g}}_\textrm{h}$ the units for both are ms$^{-2}$. **b** responses of the sickness-tuned model state estimates

To further illustrate between-model differences, the perception dynamics of the perception and sickness-tuned perception models are estimated in the frequency domain for all relevant inputs and outputs. These results illustrate how the SOMs resolve the gravito-inertial ambiguity. Figure 6 shows Bode magnitude responses for small input accelerations for both the perception-tuned and sickness-tuned models. Each subplot of the figure is referred to by its row and column number in the form $R_y C_x$.

In Fig. 6a, $R_1C_1$ and $R_1C_2$ show the horizontal plane translational acceleration estimates for the perception-tuned model as having high-pass dynamics for both the PFM and the MSOM, whereas for the SVM, this response is band-pass. For vertical acceleration inputs (as shown in $R_3C_1$), the PFM and the MSOM have a frequency-invariant constant gain for the estimated vertical acceleration. This is contrary to the SVM, which again shows a band-pass response. In the three models, the acceleration and the gravity estimates are coupled. Horizontal-plane translational accelerations (shown in $R_2C1$ and $R_2C_2$) lead, in all three models, to a low-pass response in the gravity estimate. This means that in all three models, sustained horizontal acceleration is perceived as sustained rotation. Here, the higher the perturbation frequency, the less it disturbs the perceived gravity vector. The low-pass behaviour is stronger for the SVM with a slope decreasing at a factor of 100 per decade; the PFM and MSOM are next with a slope decreasing at an approximate factor of 10 per decade. The SVM and the PFM both have their break frequency at 0.2 Hz; the MSOM has its break frequency at 0.6 Hz. Vertical accelerations do not cause a change in the vertical estimate of gravity for the MSOM and PFM (hence they cannot be shown in $R_3C_2$). The dynamics of this estimated vertical component of gravity differ for the SVM, where the SVM predicts the same low-pass response as in the horizontal plane. Horizontal plane accelerations lead to a high-pass angular velocity estimate for the PFM only. The other perception-tuned models do not include such a cross estimate of angular velocity.

Figure 6b shows the sickness-tuned responses. The acceleration estimate is of higher gain, with a larger plateau, for all the models. Likewise, the break frequencies for the gravity gain with respect to horizontal plane accelerations have shifted to lower frequencies than for the perception-tuned case. Just as in the perception-tuned case, both the MSOM and PFM are frequency-invariant for vertical acceleration perception. Interestingly, there is a qualitative difference in the frequency response for the MSOM across all responses. This takes the form of a peak in the response at approximately 0.02 Hz. This peak is most pronounced for the angular velocity estimation (shown in $R_4C1$ and $R_4C2$). A similar peak is also observed for the sickness-tuned PFM. Here there is band-pass behaviour with a maximum at $\approx$ 0.1 Hz.

### Fit to empirical sickness frequency sensitivities

**Fig. 7:**
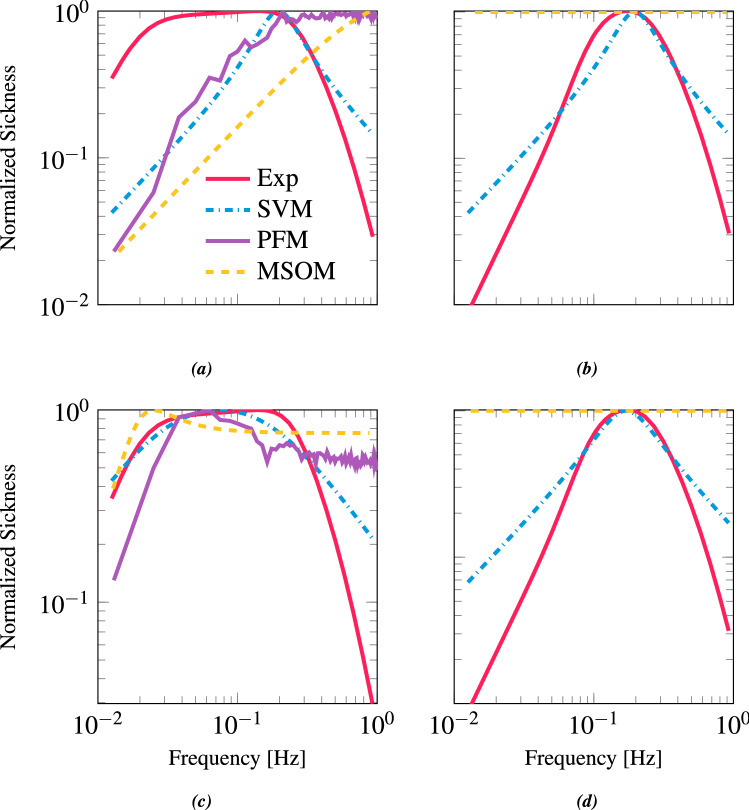
Frequency sensitivity of motion sickness to sickening stimuli. **a** Perception-tuned frequency sensitivity to horizontal plane accelerations. **b** Perception-tuned frequency sensitivity to vertical plane accelerations. **c** Sickness-tuned frequency sensitivity to horizontal plane accelerations. **d** Sickness-tuned frequency sensitivity to vertical accelerations. The red line is experimentally observed frequency sensitivity, blue line is SVM, yellow line is MSOM and magenta line is PFM. The PFM is absent in **b**, **d** because it does not have any conflicts in the vertical direction

Figure 7 shows both the perception- and sickness-tuned frequency sensitivity of motion sickness. For the MSOM and the SVM, the weights for the available conflict terms were found by fitting the empirical sickness frequency sensitivities for vertical and lateral accelerations, see Sect. 3.2. These weights are listed in Table 3 where both for the MSOM and SVM only one conflict term was selected.

Figure 7a shows the perception-tuned frequency sensitivities to lateral accelerations. Here, none of the models are seen to capture the band-pass nature of the experimental response. For Fig. 7b, only the SVM is able to fit the experimentally observed frequency sensitivity to vertical accelerations, albeit with a shift in its maximum to 0.2 Hz rather than 0.16 Hz. The MSOM shows a unit gain throughout, confirming the analytical findings in Sect. 2.1.2. Due to the absence of any angular velocity estimation, the PFM does not produce a conflict for vertical sickness and hence no sickness is predicted.

The sickness-tuned result in Fig. 7c shows that the SVM provides the closest fit to the experimental results, forming a band-pass response similar to the experimentally observed response to lateral accelerations. Likewise, the PFM and MSOM also show band-pass responses. However, unlike the SVM, the peak conflict occurs briefly at 0.06 Hz and 0.02 Hz, respectively. Lastly, Fig. 7d shows the sickness-tuned frequency sensitivity to vertical accelerations. Here, the SVM’s response is now centred on 0.16 Hz. Just as before, the MSOM has a unit gain. Lastly, as there is no angular velocity estimate induced by vertical oscillations, the PFM does not predict any sickness.

## Sickness validation

After the tuning process, the three perception models were validated for the magnitude of sickness they predict for five frequently used motion paradigms in sickness research. The selected paradigms are VTA, LTA, PRP, OVAR, and CCCP. This validation was done for both the perception tuning and the sickness tuning.

Figure 9 shows the predicted sickness for these motion paradigms for each model, alongside experimental observations. Conflicts with zero weights as found in the sickness fitting procedure are not shown. Figure 9a shows the perception-tuned sickness predictions; Fig. 9b shows sickness-tuned sickness predictions. Model performance can be evaluated by comparing the predicted sickness with the empirical data (see Sect. 2.4), which is plotted in the form of red diamonds in each panel of Fig. 9.

### Subjective vertical model

The conflict used by Bos and Bles (1998) to characterize motion sickness in the SVM was the gravity conflict. Indeed, from our sickness fitting, we confirm it to take the greatest weighting. In Fig. 9a, OVAR dominates this conflict and is 2.6 times greater than CCCP. The PRP is also seen to be of the same order as LTA and VTA, this is also a higher sickness prediction than the relative differences inferred in Sect. 2.4.

**Fig. 8:**
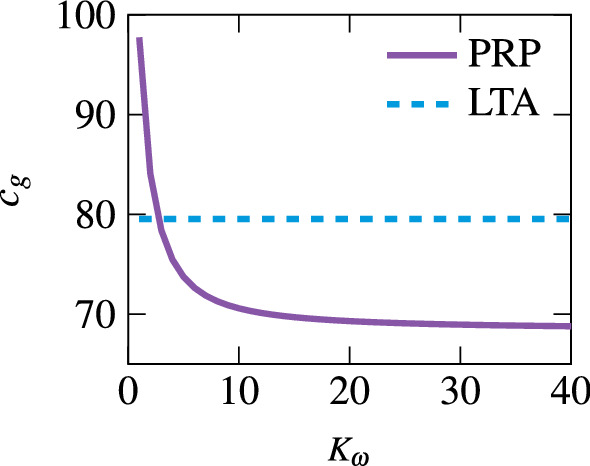
SVM gravity conflict $\vec {c}_\textrm{g}$ for PRP (purple line) and LTA (dashed blue line) with respect to angular velocity feedback gain $K_{\omega }$

**Fig. 9:**
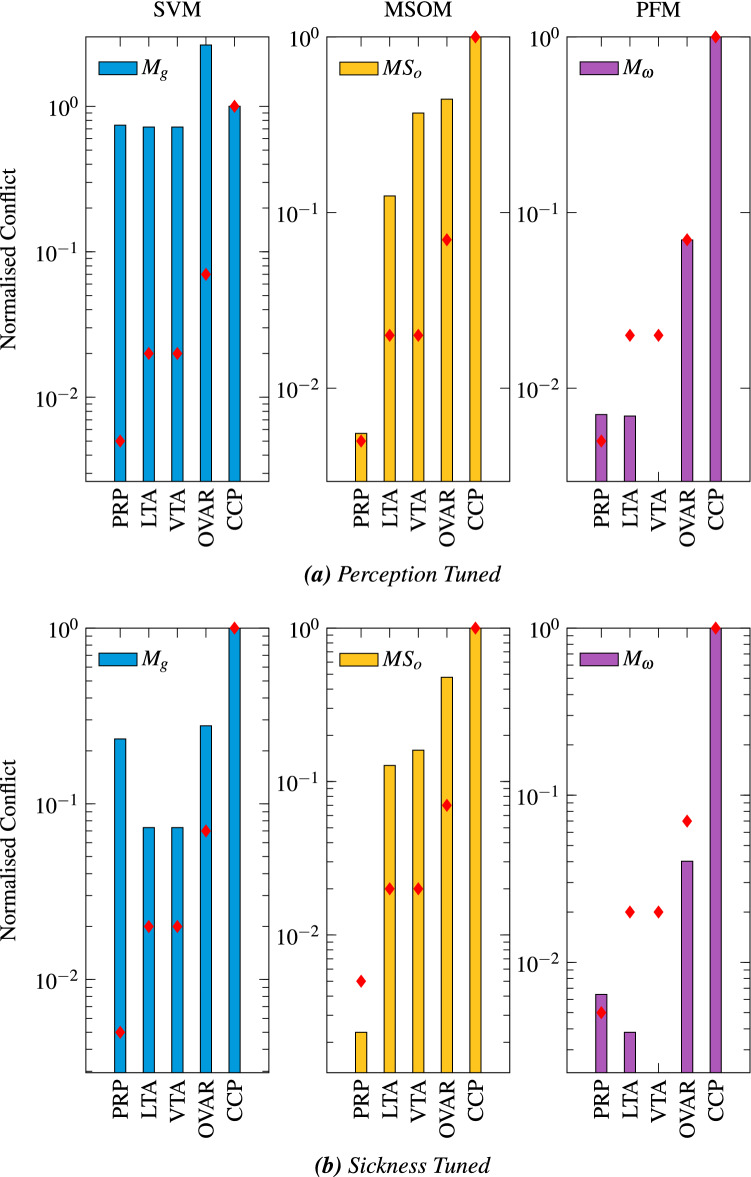
Magnitude of cumulative conflict for five motion paradigms when normalized against CCCP for the three perception models. The left column shows results for the SVM (in blue); the middle columns show results for the MSOM (yellow); and the right column shows the results for the PFM (magenta). The top row **a** shows predicted sickness for perception-tuned models; the bottom row **b** shows the predicted sickness for sickness-tuned models. Red diamonds mark experimental results

The sickness-tuned SVM follows the empirical observations more closely (see Fig. 9b). Here, unlike for the perception-tuned case, the relative magnitudes of CCCP, OVAR and LTA approximately match the expectations. One difference again is the greater strength of PRP. The reason for this consistent over-estimation is due to the role of the angular velocity feedback. In the SVM, the gain $K_\omega$ works to track the sensed signal. A weak angular velocity feedback means that there is a greater difference between the sensed and the internally estimated angular velocity. As the angular velocity is used in the form of the Mayne equation (Mayne 1974) to rotate the gravity vector, there is a greater difference between the sensed gravity and internally estimated gravity, i.e. the gravity conflict. Increasing the angular velocity feedback gain reduces this conflict. Figure 8 shows this variation in gravity conflict with respect to both PRP and LTA as a function of $K_{\omega }$.

**Table 3:** Weights on the different conflict terms found by fitting the empirical sickness frequency sensitivities

Models	Conflicts				
	$c_{\omega }$	$c_\textrm{g}$	$c_\textrm{a}$	$c_\textrm{o}$	$c_{\textrm{oa}}$
MSOM	0	n/a	n/a	1	0
SVM	0	1	0	n/a	n/a
PFM	1	n/a	n/a	n/a	n/a

As there is no connection between the perceived rotational velocity and linear acceleration estimates for the SVM, for LTA it is unresponsive to changes in $K_{\omega }$. Interestingly, for PRP the conflict asymptotically converges to around 68 ms$^{-2}$/rad, which is only marginally smaller than the value found for LTA and so does not match the experimental findings (Fig. 8). Moreover, for $K_{\omega } \ge 4$ the EVAR time constant is $> 28$ s, meaning that even if the conflict is reduced, the velocity storage time constant leaves the bounds of experimentally observed angular velocity perception values.

### Multi-sensory observer model

For the MSOM, the perception-tuned otolith magnitude error in Fig. 9a shows that CCCP, OVAR and LTA follow a reasonable ranking of sickness intensity. There is, however, a large difference between the predicted sickness magnitude for VTA and LTA, which is contradicted by experimental data. The reason for this is the flat nature of the frequency response (Fig. 7b) for VTA compared to the LTA (Fig. 7a), which attenuates the sickness due to lateral accelerations. The predicted sickness for PRP is close to experimental findings. This is because the MSOM is able to easily discriminate the roll signal from acceleration, leading to only a small sensory conflict.

The sickness-tuned MSOM makes similar predictions to the prediction-tuned MSOM. Here, the CCCP is again confirmed to be the most sickening, followed by OVAR. Still, the magnitude of the difference between CCCP and OVAR is not similar to experimental findings. The sickness-tuned responses for VTA and LTA are of similar amplitude. This is because the frequency responses to vertical and lateral accelerations as shown in Fig. 7b, c are more similar to each other at the frequency of 0.2 Hz, which was the input perturbation frequency for the validation. Likewise, similar to the perception-tuning, the sickness-tuned model is able to discriminate the roll signal from acceleration, leading to a negligible roll-induced sensory conflict.

### Particle filter model

Due to lack of otolith or other acceleration conflict, the PFM predicts no motion sickness for the vertical motion paradigm. For the rotational motion paradigms, however, both the perception-tuned and the sickness-tuned PFM perform well. OVAR and PRP are found to be a factor of 27 and 170 times smaller in sickness magnitude than CCCP, which matches well with the experimental values (of 14 and 200 times, respectively). The reason why the angular conflict is larger in OVAR than in PRP for the PFM is the existence of a strong oscillation in the estimated angular velocity in both fore-aft and lateral directions. This effect is not present in the SVM due to the lack of a bidirectional influence between the otoliths and the semicircular canals, and it is attenuated in the MSOM due to the gain $K_{f\omega }$ being set to zero to optimize perception predictions.

## Discussion

Motion sickness has long been linked to state estimation conflicts in motion perception. In this study, we evaluated different perception models for their ability to reproduce both empirical motion perception and motion sickness data. The models were tuned to time-domain perception data, as well as frequency-domain sickness data. Evaluating both the perception-tuning and sickness-tuning results together helped identify whether the SOM framework is effective for predicting motion sickness.

The perception-tuned SVM, MSOM and PFM could all adequately approximate most selected perceptual phenomena, but for each there were also important deviations from measured data from literature. The sickness-tuned models on the whole did not adequately fit the perception data. Likewise, the success of fitting to sickness frequency sensitivities was limited (Fig. 7), with only the SVM providing reasonable fits. In the following, we first discuss the ability of the different models to reproduce human motion perception, and then discuss their ability to reproduce observations on motion sickness. In the latter discussion, we specifically evaluate the role of sensory conflict and identify two key mechanism that may be required in any model of motion sickness.

### Perception

Overall, the perception-tuned models described empirical perception data better than the original untuned models (SMAE of 0.243 compared to 0.354, indicative of smaller residual variance in the former). This is expected because the tuning was done with respect to a different collection of paradigms than the disparate paradigms each individual model was tuned on in their original publications. The sickness-tuned models on average had an SMAE of 0.418, indicating that both sickness and perception phenomena could not be captured by the same set of parameters. The implication of this is that current models of perception are not directly suitable for predicting motion sickness.

#### Subjective vertical model

For the perception tuning, the time-domain perception predictions of the SVM were found to be accurate. The model was able to satisfactorily match the empirical EVAR, OVAR and centrifugation responses. Interestingly, for centrifugation, the yaw rate of the centrifuge had a profound impact on the model predictions. However, there is no clear effect of yaw velocity in the experiments evaluated. For instance, in Curthoys (1996) the time constant of the subjective vertical was 6.1 s at $200^\circ \,\textrm{s}^{-1}$, whereas this was 14.0 s for Merfeld et al. (2000) at $250^\circ \,\textrm{s}^{-1}$ and 15.9 s for Graybiel and Brown (1951) at $60^\circ \,\textrm{s}^{-1}$. To match the experimental data, the observer gains of the SVM need to be dependent on the yaw rate. This means that there were many parameter sets that approximately satisfied the error minimization criterion when all centrifugation conditions were optimized together. Therefore, the model in its current form is over-parametrized with respect to the perception predictions for the data broad range of data analysed in this study. Moreover, in the frequency domain, the estimated acceleration for all directions has band-pass dynamics. However, it is known from literature that the dynamics should be high-pass (Merfeld et al. 2005a, b).

#### Multi-sensory observer model

As it has the smallest overall error, the time-domain perception-tuned perception predictions of the MSOM are the most accurate. It is important to mention that the gain $K_{f\omega }$, responsible for the coupling between the semicircular canals and the otoliths, was set to 0 in the perception-tuned MSOM, compared to the value of 8 in the original model. This is due to the reports of zero angular velocity perception in the experiments of Vingerhoets et al. (2005) on OVAR. Moreover, just like the SVM, there is no unique set of parameters that describes all centrifugation results. In the frequency domain, the acceleration and gravity estimates of the MSOM for horizontal plane acceleration input match literature findings. Interestingly, the vertical acceleration percept is a unit gain. This means the vertical acceleration estimate is frequency invariant. Studies indeed suggest that there is anisotropy in our perception of travelled distance, with vertical translations being worse than horizontal plane translations (Hinterecker et al. 2018). It is not clear from literature, however, whether this anisotropy is caused by differences in the frequency response of our perception to vertical acceleration or indeed due to the differences in the integration of acceleration into travelled distances by the central nervous system. Experiments on the frequency response of vertical displacement perception may help validate the model for this degree of freedom.

#### Particle filter model

In the PFM, there is a trade-off between fitting the OVAR and centrifugation responses. Decreasing the standard deviation of the acceleration prior, $\sigma _\textrm{a}$, led to more accurate centrifugation responses, but also increased the yaw angular velocity bias-term on the OVAR responses. The bias in the OVAR is present in the PFM because reducing the standard deviation of the prior on acceleration makes perceiving a translation along a cylindrical trajectory during OVAR less likely than rotating in yaw whilst tilting, whereas experiments (Angelaki and Yakusheva 2009) show that during long exposure to OVAR only a translational percept remains and heading is perceived to be constant. In the frequency domain, the responses of the PFM and the MSOM are, despite employing fundamentally different methods, very similar. This is because both models make the key assumption that the magnitude of the gravity vector is constant and, unlike the SVM, perform no pre-filtering.

### Sickness

For all three models, the sickness tuning provided a better fit to motion-sickness data, as expected. Out of the compared models, the SVM, with a combined SMAE of 0.074, provided the closest fit to both the lateral and vertical sickness frequency responses reported in the literature. In comparison, the SMAE was 0.259 for the MSOM and 0.343 for the PFM, respectively.

The validation data set was used to evaluate the predicted relative sickness magnitude across a broader range of motion conditions. In this, the validation results for the different models were more mixed. Although the PFM gave the best performance for the rotational degrees of freedom, it did not predict any vertical sickness. In comparison, both the SVM and the MSOM made better predictions for motion sickness due to inertial accelerations. For the SVM there was an overestimation of the sickness resulting from roll rotations, whereas for the MSOM roll rotation did not lead to any sickness. With respect to the difference between OVAR and CCCP, both sickness tuned models performed similarly.

#### Subjective vertical model

Out of the models compared, the best match between the empirical frequency-sensitivity data for sickness and model predictions was obtained for the SVM model. Inspection of the fitting results revealed that the model when tuned to sickness-fitted VTA responses remarkably well, better than the other models. The underlying reason for why the SVM is the only model capable of frequency-variant VTA-induced sickness predictions is that the magnitude of the estimated gravity is permitted to vary. Here, there is a leakage from the gravito-inertial force to the magnitude of the estimated gravity term. For both the MSOM and the PFM, the magnitude of gravity is fixed to 9.81 ms$^{-2}$. In hypergravity experiments involving centrifugation such a fixed magnitude of gravity predicts a perceived sense of translation, however, this is not observed (Clark et al. 2015). This supports the notion that the internally expected magnitude of gravity is variable, as allowed in the SVM.

Despite its success, one drawback of the SVM is that it is a partial-observer. Specifically, head angular velocity is observed, but acceleration and gravity are not. Instead, the gravito-inertial force is first low-pass and then high-pass filtered. The output of the filters are the sensed gravity and acceleration, respectively, which are tracked by the internal model. This direct filtering of the gravito-inertial force has the most pronounced influence on the model’s output. The resulting tracking error directly leads to the conflict terms. This mechanism is an important theoretical deviation from the general observer framework outlined by Oman (1982). This explains the reduced accuracy for predicting motion sickness for rotational motions, (see Fig. 9b) as is evident by the SVM’s high sickness predictions for PRP, which contradict experimental data (shown in Fig. 8).

#### Multi-sensory observer model

When tuned for sickness, the MSOM somewhat approximates the band-pass dynamics that are associated with motion sickness caused by horizontal plane accelerations (see Fig. 7c). It is notable that the relevant conflict is generated only for the somatogravic effect. The somatogravic effect is a striking demonstration of a perceptual conflict and occurs when there is an ambiguity in the sensation of tilt and translation during low-frequency horizontal accelerations. Previous research, including our own, has supported it being a possible contributor to motion sickness (Wood 2002; Wood et al. 2007; Irmak et al. 2021c). It is a positive outcome that there is a direct perceptual correlate of this conflict term in the MSOM. Though the SVM does model the somatogravic effect, due to the absence of an explicit two-way coupling between the semicircular canals and the otoliths, there is no difference in the frequency sensitivities between vertical and horizontal, unless parameters are tuned explicitly for the different directions, as done in this study. Moreover, because unlike the SVM it is a full observer, the MSOM is able to accurately discriminate roll rotation, which makes the predicted motion sickness from roll closer to the experimentally observed values (Fig. 9b).

The main disadvantage of the MSOM is that the peak predicted frequency of sickness for LTA is at 0.02 Hz. It is, however, known from experiments (Donohew and Griffin 2004) that that sickness occurs over a broader range of frequencies for LTA and peaks at a higher frequency (0.03 - 0.3 Hz). Moreover, the MSOM results in frequency-invariant vertical motion sickness predictions (see Fig. 7d). This is mostly not observed in previous experiments (Zaichik et al. 1999) and is due to the nature of the acceleration estimation process, which assumes a constant magnitude for the gravity vector.

#### Particle filter model

Similar to the MSOM, the PFM includes/provides band-pass sickness predictions through the somatogravic effect mediated by the two-way coupling between the semicircular canals and the otoliths, thus providing a perceptual correlate for its conflict term. Interestingly, the PFM provides the most accurate fit to motion sickness due to rotations (see Fig. 9b). This may be due to the fact that it can account for the nonlinear dynamics introduced by them. Moreover, unlike the other two models, it is a probabilistic model that performs Bayesian inference on vestibular stimuli. This is advantageous because it automatically accounts for the presence of noise at the input, which the other models do not. Not only are probabilistic models a more faithful representation of reality, but they may also explain non-classical behaviour. For instance, it is known that noisy galvanic vestibular stimulation (GVS) as well as bone conducted vibrations (BCV) retard the development of motion sickness (Weech et al. 2018). Probabilistic approaches may help explain this behaviour in an emergent fashion, as a consequence of performing inference on more noisy sensory afferents. In contrast, deterministic models such as the SVM and the MSOM may only be fitted to the data manually and provide no deeper explanatory power.

The key disadvantage of the PFM is that it does not predict any VTA-induced sickness. This is because, unlike the MSOM, there is no internal estimate for the gravito-inertial force. Moreover, the model performs inversion of the semicircular canal signal $C_t$ to obtain an estimate of the head velocity $\omega _t$. Inverse models are affected more by noise than forward models, and for some problems the solution space may even be infinite. This makes employing forward models more appealing.

#### Implications for sickness modelling

The current study shows that SOM parameters fitted to perception data do not accurately predict motion sickness and vice versa. A combined fitting may of course yield a compromise, but will not address the fundamental limitations identified in this study. Firstly, the MSOM and PFM have a contradictory or even an absent prediction of sickness for vertical acceleration stimuli. Moreover, the SVM predicts a higher magnitude of motion sickness for roll than is observed empirically, whilst deviating substantially from the theoretical framework developed by Oman (1982).

We propose that a more complete model of motion sickness would contain the following two mechanisms identified in this study. Firstly, models should include an explicit two-way coupling between the semicircular canals and the otoliths (Lim et al. 2017), which is essential for modelling the somatogravic effect. In our study, we found that the somatogravic effect explains sickness under horizontal plane accelerations in the PFM and MSOM. Moreover, the inclusion of such a two-way coupling is required to have qualitatively different vertical and horizontal sickness dynamics for the SVM. Without this coupling, vertical and horizontal sickness dynamics are incorrectly assumed to be the same (Wood 2002; Wood et al. 2007; Irmak et al. 2021c). Secondly, models should have a variable estimate for the magnitude of gravity. Without allowing variability in the magnitude estimate, it is impossible to capture how vertical motion, as it does not result in any perceived rotations, can lead to motion sickness.

Furthermore, the underlying framework in which these two mechanisms are implemented should be probabilistic. For this, a reformulation of the PFM is needed whereby only forward models of sensory processing are used. Moreover, the PFM has frequency-invariant likelihoods, whereas humans are known to have frequency-dependent likelihoods (Angelaki et al. 2009). Specifically, low-frequency stimulation of the otoliths is more likely to be due to tilt, while high-frequency stimulation is more likely to be due to translation. Angelaki et al. (2009) state that the system *“knows”* what these likelihoods should be. The system can indeed *learn* the frequency-dependent likelihoods by matching vestibular stimulation patterns to a ground truth. In experimental settings, this ground truth would be defined by the researcher, but in reality it would likely become implicitly evident to the organism via the visual and proprioceptive cues. The likelihoods would be used to estimate the real states of the organism and the expected sensory stimulation, which would then be compared with actual sensory stimulation to drive sensory conflict terms. The sensory conflict terms would be used concurrently to both update the internal model and also to drive motion sickness. The probabilistic nature of the conflict generation process would not only allow for the modelling of the small but evident variance in intra-individual sickness trajectories seen in our previous work (Irmak et al. 2021b), but could also account for other emergent phenomena such as the effect of increased sensory noise via GVS, BVC or even ageing (Nestmann et al. 2020).

Beyond presenting vestibular-only data in this study, the authors also attempted to include vision in the models evaluated. For the SVM, the model proposed by Bos et al. (2008a) was taken. For the MSOM the full visual-vestibular formulation by Newman (2009) was used. Lastly, the form of the PFM formulated in Laurens and Droulez (2008) was used. Visual inputs accounted for in the SVM and MSOM include angular and linear velocity, as well as orientation, whereas for the PFM only angular velocity is included. As observed in experiments (Irmak et al. 2021b), including vision decreases sensory conflict and consequently, motion sickness. Regardless, vision was ultimately omitted in this study. This was primarily because the benefits of including vision were outweighed by the complexity introduced by the increased number of parameters and the lack of sufficient experimental results in the literature to define and validate models of visual contributions to motion perception. Nonetheless, the effect of vision remains a crucial focus for future work and is currently being pursued by researchers (Bos et al. 2008b; Yunus et al. 2022; Wada et al. 2020).

In this study, a simple integrator was used to model the accumulation of motion sickness over time. In reality, this accumulation is more complex, with both time and amplitude nonlinearities. For instance, it is known that as humans become sick, their sensitivity to further sickness increases (Oman et al. 1986; Golding and Stottt 1997; Irmak et al. 2021b). Moreover, there may be individual differences in the temporal dynamics of sickness (Irmak et al. 2022). Lastly, motions below a certain amplitude may often fail to elicit any sickness (Lawther and Griffin 1988). These effects should be accounted in a complete model of motion sickness.

### Future work

To bring clarity to motion sickness modelling via the sensory conflict approach, additional experimental data regarding perceived acceleration (or translation, as the more easily measured correlate) and gravity (or tilt) frequency responses as analysed in this paper should be obtained. These should be directly related to the sickness frequency responses observed under the same motion paradigms. This would help to identify the nature of the relationship between the somatogravic effect and motion sickness.

Recent experiments also show major differences between participants in both perception and motion sickness (Irmak et al. 2021c). As these two are coupled per the sensory-conflict approach, we can use SOMs to quantify individual variations in sickness sensitivity through observer parameters (e.g. feedback gains). For instance, Dai et al. (2010) used their SOM to relate eye velocities and motion sickness observed during OVAR. Here individualization is important, as per the “Ecological Fallacy” averaging over an entire group may mask important relationships between sickness and perception that occur at the individual level. Indeed, our previous work (Irmak et al. 2021c) has shown that individuals have differing frequency sensitivities and so fits to population averaged sensitivities are not always appropriate.

For validation of future and current models, investigation of sickness across a wide range of sickening phenomena is needed. This study attempted such a validation in Section 4, but in the absence of experiments employing a consistent sickness scale such as the MIsery SCale (Bos et al. 2005) and a consistent perturbation duration and magnitude, unfortunately, only approximate comparisons could be performed. Having extensive combined perception/sickness data would allow us to better discriminate between competing models and allow identification of causal mechanisms.

Lastly, the models evaluated in this study were all without active control, and so ‘open-loop’, i.e. only informed by sensory stimuli. Experiments by Riccio and Stoffregen (1991) have shown that orientation perception, rather than being open-loop as modelled here, may in fact be a closed-loop process. This means that the vertical is perceived with respect to the “Direction of Balance”. This would have obvious consequences for modelling sickness, which we believe to be downstream of perception and thus, under this framework, action. However, later studies (Panic et al. 2015) have found that perception of vertical is not informed by the direction of balance and so open-loop modelling is sufficient to describe orientation perception. These two contradictory studies highlight the need for further investigation. And Indeed, given the negative findings of the current study as per the models evaluated, a closed-loop framework of joint action-perception may yield better results and perhaps even help identify the relationship between the sensory conflict and postural instability theories, and thus potentially unify both interpretations.

## Conclusion

The results indicate that all models are able to provide good perception fits to the selected motion paradigms. Despite this, there are large differences in the frequency response of the acceleration and gravity perception between the SVM, the MSOM and the PFM. This is attributed to the different assumptions made for resolving the gravito-inertial ambiguity in each model. Under the observer framework for sickness prediction, these assumptions are found to directly influence the sickness predictions.

Overall, none of the models can capture the full range of empirical motion sickness observations considered in this study, including lateral and vertical translational acceleration, pure roll, off-vertical axis rotation and cross-coupled coriolis perturbations.

Based on our model comparisons, we identified two critical components that may resolve this. The first mechanism is the coupling of the semicircular canal with the output of the otoliths to compute a more reliable gravity (i.e. orientation) and acceleration estimate. This is necessary to capture the differences in the dynamics between motion sickness induced by horizontal and vertical accelerations. The second mechanism is that the models should have a variable estimate for the magnitude of gravity. Without a slowly varying magnitude estimate, models cannot predict the well-known motion sickness due to vertical accelerations. In addition, we propose that these mechanisms are best implemented within in a probabilistic framework similar to the particle filter evaluated in this study. Adoption of such a framework will better account for individual differences in sickness susceptibility, as well as emergent phenomena such as variations in sickness sensitivity caused by changes in sensory noise induced by GVS/BCV of the mastoids. This makes the approach the most promising research direction towards key applications for mitigating motion sickness in automated vehicles.

## Figures and Tables

**Fig. 10:**
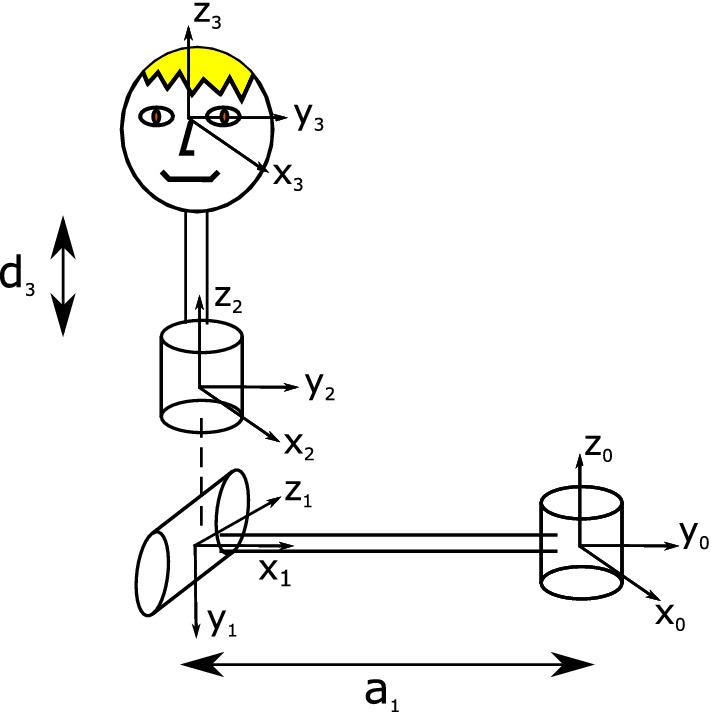
Kinematic chain used for complex motion generation

## Motion implementation

For this purpose, the following kinematic chain shown in Fig. 10 is used

Here ${\textbf {R}}_0$ defines the earth fixed coordinate system, composed of the unit vectors $\vec {X_0}, \vec {Y_0}, \vec {Z_0}$. Likewise, ${\textbf {R}}_3$ defines the head fixed coordinate system. Using the standard Denavit–Hartenberg (DH) formulation, one can derive the set of transforms that map ${\textbf {R}}_3$ to ${\textbf {R}}_0$ and likewise ${\textbf {R}}_0$ to ${\textbf {R}}_3$ or any therein between. The DH parameters for this kinematic chain are tabulated in Table 4

**Table 4:** DH parameters for kinematic chain

Link	$\alpha$	a	d	$\theta$
1	$-\pi /2$	$a_1$	0	$\pi /2 + \theta _1$
2	$\pi /2$	0	0	$\theta _2$
3	0	0	$d_3$	$\theta _3$

The parameters $a_1$ and $d_3$ are to specify the centrifugation distance and the distance of roll point to the vestibular organs, respectively. For centrifugation, $a_1$ is simply defined as the distance of the body from the centrifugation axis. $d_3$ depends on the nature of the motion. If the motion is such that the roll rotation at the pivot point ${\textbf {R}}_1$ is about the vestibular organ, as is the case for OVAR, then $d_3$ is set to zero. If however the roll point is the neck, as is the case for cross-coupled coriolis, $d_2$ is set to the distance between the neck and the vestibular organs. As a result, the transform which relates the head frame to the earth frame is given by

13 $$\begin{aligned} {\textbf {T}}^{0}_3 = {\textbf {T}}^{0}_1 {\textbf {T}}^{1}_2 {\textbf {T}}^{2}_3 \end{aligned}$$

This transform takes the general form

14 $$\begin{aligned} {\textbf {T}}^{0}_3 = \begin{bmatrix} r_{11} &{} r_{12} &{} r_{13} &{} d_x \\ r_{21} &{} r_{22} &{} r_{23} &{} d_y \\ r_{31} &{} r_{32} &{} r_{33} &{} d_z \\ 0 &{} 0 &{} 0 &{} 1 \\ \end{bmatrix} \end{aligned}$$

Where the elements of the matrix are given as

15 $$\begin{aligned} \begin{array}{l} r_{11} = -c_1 s_3 - c_2 c_3 s_1 \\ r_{12} = c_2 s_1 s_3 - c_1 c_3 \\ r_{13} = -s_1 s_2 \\ r_{21} = c_1 c_2 c_3 - s_1 s_3 \\ r_{22} = -c_3 s_1 - c_1 c_2 s_3 \\ r_{23} = c_1 s_2 \\ r_{31} = -c_3 s_2\\ r_{32} = s_2 s_3\\ r_{33} = c_2 \\ d_x = -s_1(a_1 + d_3 s_2) \\ d_y = c_1(a_1 + d_3 s_2)\\ d_z = d_3c_2 \end{array} \end{aligned}$$

Here for the sake of brevity and legibility $sin(\theta _1)$ and $cos(\theta _1)$ is denoted as $s_1$ and $c_1$ respectively. ${\textbf {T}}^{0}_3$ relates the head frame to the earth frame, its inverse relates the earth fixed frame to the head frame. The inverse is equivalent to the transpose of the original transform. Therefore

$$\begin{aligned} {\textbf {T}}^{3}_0 = ({\textbf {T}}^{0}_3)^{T} \end{aligned}$$

With this one can transform earth referenced gravity to the head coordinates to simulate what the gravity component of the gravito-inertial force would be.

$$\begin{aligned} \vec {g}_{3} = {\textbf {T}}^{3}_0\vec {g}_{0} \end{aligned}$$

The semicircular canals report angular velocity with respect to the head. To acquire the head referenced angular velocity the Jacobian must be evaluated in the head frame of reference. This yields

$$\begin{aligned} {\vec {\nu }_3} ={\textbf { J}}_3(\vec {\theta }){\vec {\dot{\theta }}} \end{aligned}$$

Where

$$\begin{aligned} \vec {\nu }_3 = \begin{bmatrix} v_x \\ v_y\\ v_z \\ \omega _x\\ \omega _y\\ \omega _z\\ \end{bmatrix} \end{aligned}$$

In the head frame. As this is the head frame, the velocity terms are zero and so only the angular velocity terms are left. These can be used straight away as inputs to the semicircular canals.

Similarly, by expressing the Jacobian in the earth frame, differentiating the linear velocity and transforming back to the head frame one can derive the linear head acceleration in the head frame.

$$\begin{aligned} \vec {\nu }_0 ={\textbf { J}}_0(\vec {\theta })\vec {\dot{\theta }} \end{aligned}$$

This gives linear velocity in the earth frame. Numerically differentiating

$$\begin{aligned} \vec {a}_0 = \frac{\vec {v}_0(t + \delta t) - \vec {v}_0(t-\delta t)}{2\delta t} \end{aligned}$$

gives the linear acceleration in the earth frame, where $\delta t$ is the differentiation time step used. In this case, a second order numerical technique is used. The head acceleration in the earth frame is then transformed in much the same way as the gravity, in the head frame.

$$\begin{aligned} \vec {a}_{3} = {\textbf {T}}^{3}_0\vec {a}_{0} \end{aligned}$$

Therefore, the gravito-inertial acceleration in the head frame $f_3$, as sensed by the otoliths is given by

$$\begin{aligned} \vec {f}_{3} = \vec {g_3} - \vec {a_3} \end{aligned}$$

This can be used straight away as the input to the otolith model.

## Conflict power

Assume a conflict of magnitude A and angular velocity $\omega _p$

$$\begin{aligned} |c(t)| = |A\sin {\omega _p t}| \end{aligned}$$

The Fourier series expansion yields the following DC ($A_0$) and harmonic amplitude ($A_n$) terms,

$$\begin{aligned}{} & {} A_0 = \frac{2|A|}{\pi } \\{} & {} A_n = \frac{4|A|}{\pi (n^2 - 1)} \end{aligned}$$

Where $$n = 2, 3, 4 \dots $$

Therefore, the conflict signal has most of its power in the DC bias and the first harmonic terms. The second-order system applied to accumulate sickness filters the signal. The gain for this is given as

$$\begin{aligned} G = \frac{K}{(1 + \omega \mu )^2} \end{aligned}$$

Therefore the end magnitude of the bias and first harmonic of the filtered absolute conflict signal is

$$\begin{aligned} A_0 = \frac{2|A|K}{\pi } \\ A_n = \frac{4|A|K}{3\pi (1 + 4\omega _p \mu )^2} \end{aligned}$$

For a constant value of |*A*| the lower the frequency of conflict the larger the sickness response, due to the contribution of harmonics. The time constant $\mu$ used in Bos and Bles (1998) is 12 minutes and K is equal to 0.85. These free parameters depend on the sickening motion, which for them was pure vertical. A smaller time constant allows for the higher harmonics to contribute, but as observed, there is quadratically decreasing energies with respect to harmonic position. More importantly, the assumption of constant |*A*| is invalid. Accurate prediction of sickness requires smaller sickness at lower and higher frequencies, therefore one can expect a parabola in the frequency domain with centre frequencies ranging between 0.03 and 0.3 Hz. At such frequencies, for the first harmonic to not be filtered by the second-order system, the time constant must be around 30 s. Such small time constants may be observed for cross-coupled Coriolis stimulation and so the frequency of the conflict may become important here, i.e. there may be substantial harmonics added on to the base increase in sickness (waves of nausea with each head rotation). However, for the longer time constants observed in the majority of cases the first harmonic does not contribute at all and only the DC bias is important.

## SVM lateral and vertical response derivation

Here, take only one perturbation direction. As there is no cross-coupling between the input and output direction, one can do the analysis on a given component, *f* of the gravito-inertial force vector $\vec {f}$.

$$\begin{aligned}{} & {} f = \frac{K_\textrm{a}}{s} + \frac{K_\textrm{g}}{s}c_\textrm{g} \\{} & {} c_\textrm{a} = f\frac{\tau _{\textrm{lp}}s}{\tau _{\textrm{lp}}s + 1} - {\hat{f}}\frac{\tau _{\textrm{lp}}s}{\tau _{\textrm{lp}}s + 1} \\{} & {} c_\textrm{g} = f\frac{1}{\tau _{\textrm{lp}}s + 1} - {\hat{f}}\frac{1}{\tau _{\textrm{lp}}s + 1} \\{} & {} c_\textrm{a} = f\frac{\tau _{\textrm{lp}}s}{\tau _{\textrm{lp}}s + 1} - \Big (\frac{K_\textrm{a}}{s}c_\textrm{a} + \frac{K_\textrm{g}}{s}c_\textrm{g}\Big )\frac{\tau _{\textrm{lp}}s}{\tau _{\textrm{lp}}s + 1} \\{} & {} c_{\textrm{a}}s + c_{\textrm{a}}\Big (K_\textrm{a} + \frac{1}{\tau _{\textrm{lp}}}\Big ) + K_{\textrm{g}}c_{\textrm{g}} = fs \end{aligned}$$

Taking the inverse laplace transform with zero innitial conditions

$$\begin{aligned} \dot{c}_{\textrm{a}} + c_{\textrm{a}}\Big (K_\textrm{a} + \frac{1}{\tau _{\textrm{lp}}}\Big ) + K_{\textrm{g}}c_{\textrm{g}} = \dot{f} \end{aligned}$$

Similarly for $$c_\textrm{g}$$

$$\begin{aligned} \tau _{\textrm{lp}}\ddot{c}_{\textrm{g}} + \dot{c}_{\textrm{g}} + K_{\textrm{g}}c_{\textrm{g}} + K_{\textrm{a}}c_{\textrm{a}} = \dot{f} \end{aligned}$$

This system of ordinary differential equations can be represented in state space form such that

$$\begin{aligned}{} & {} \vec {\dot{q}} = {\textbf {A}}\vec {q} + {\textbf {B}}\vec {f} \\{} & {} \begin{array}{cc} \vec {y} = \begin{bmatrix} c_\textrm{a}\\ c_\textrm{g}\\ \dot{c}_\textrm{g} \end{bmatrix} = {\textbf {C}}\vec {q} + {\textbf {D}}\vec {f} \end{array} \end{aligned}$$

Where $\vec {f}$ here is defined as the sole component perturbing the system. For instance, if there is only lateral acceleration

$$\begin{aligned} \begin{array}{cc} \vec {f} = \begin{bmatrix} f_y\\ f_y\\ f_y \end{bmatrix} \end{array} \end{aligned}$$

Therefore following through

$$\begin{aligned}{} & {} \begin{array}{cc} {\textbf {A}} = \begin{bmatrix} -K_\textrm{a} -1/\tau _{\textrm{lp}} &{} -K_\textrm{g} &{} 0\\ 0 &{} 0 &{} 1/\tau _{\textrm{lp}} \\ -K_\textrm{a} &{} -K_\textrm{g} &{} -1/\tau _{\textrm{lp}} \end{bmatrix} \end{array} \\{} & {} {\textbf {B}} = \begin{bmatrix} -K_\textrm{a} - 1/\tau _{\textrm{lp}}&{} 0 &{} 0 \\ 0 &{} \frac{1}{\tau _{\textrm{lp}}} &{} 0 \\ 0&{}0&{} -K_\textrm{a} - 1/\tau _{\textrm{lp}} \\ \end{bmatrix}\ \\{} & {} {\textbf {C}} = \begin{bmatrix} 1&{} 0 &{} 0 \\ 0 &{} 1 &{} 0 \\ 0&{}0&{} \tau _{\textrm{lp}} \\ \end{bmatrix}\ \\{} & {} {\textbf {D}} = \begin{bmatrix} -1&{} 0 &{} 0 \\ 0 &{} 0 &{} 0 \\ 0&{}0&{} -1 \\ \end{bmatrix}\ \end{aligned}$$

Now transforming this back one can obtain the gravito-inertial force to conflict transfer functions.

## MSOM lateral response derivation

First, the simpler system without the semicircular canals influence is derived. It is assumed that the only forcing terms are $f_y$ and $f_z = \hat{f_z} = -k$ where $b = 9.81$. Here $f_x = \hat{f_x} = 0$.

The conflict terms are defined as

$$\begin{aligned} \vec {c_\textrm{o}} = \vec {f} - \vec {{\hat{f}}} \\ \vec {c_{\textrm{oa}}} = \frac{ \vec {f}x \vec {{\hat{f}}}}{| \vec {f}|| \vec {{\hat{f}}}|} \end{aligned}$$

Let us expand the otolith angle conflict in to vector form

$$\begin{aligned} \begin{vmatrix} i&j&k \\ f_x&f_y&f_z \\ \hat{f_x}&\hat{f_y}&\hat{f_z} \nonumber \end{vmatrix} = \begin{matrix} (f_y\hat{f_z} - f_z\hat{f_y})i\\ -(f_x\hat{f_z} - f_z\hat{f_x})j\\ (f_x\hat{f_y} - f_y\hat{f_x})k\\ \end{matrix} \end{aligned}$$

As $$f_x = \hat{f_x} = 0 $$ and $$ f_z = \hat{f_z} = k$$

$$\begin{aligned} fx{\hat{f}} = -b(f_y - \hat{f_y})i \end{aligned}$$

Where all the other components are zero. As the equation above divides the cross product by the magnitude of the respective vectors this too must be done. Assuming small perturbation for $f_y$ the product of the magnitude of the two is $b^2$ Therefore the final otolith angle conflict may be written as

$$\begin{aligned} \vec {c_{\textrm{oa}}} = \frac{ \vec {f}x \vec {{\hat{f}}}}{| \vec {f}|| \vec {{\hat{f}}}|}{} & {} = -\frac{1}{k}(f_y - \hat{f_y})i , 0j, 0k \\{} & {} = -\frac{1}{k}c_{\textrm{oy}}i , 0j, 0k \end{aligned}$$

The way the magnitude error evolves can be written as

$$\begin{aligned} \vec {c_\textrm{o}} = \vec {f} - \vec {{\hat{f}}} \end{aligned}$$

Where

$$\begin{aligned} \vec {{\hat{f}}} = \vec {{\hat{g}}} - \vec {{\hat{a}}} \end{aligned}$$

Therefore

$$\begin{aligned} \vec {c_\textrm{o}} = \vec {f} - \vec {{\hat{g}}} + \vec {{\hat{a}}} \end{aligned}$$

As

$$\begin{aligned}{} & {} \vec {{\hat{a}}} = K_\textrm{a}\vec {c_\textrm{o}} \\{} & {} \vec {c_\textrm{o}} = \vec {f} - \vec {{\hat{g}}} + K_\textrm{a}\vec {c_\textrm{o}} \end{aligned}$$

Therefore the magnitude error can be written as

$$\begin{aligned} \vec {c_\textrm{o}} = (\vec {f} - \vec {{\hat{g}}})\frac{1}{1 - K_\textrm{a}} \end{aligned}$$

The derivative of this with respect to time gives

$$\begin{aligned} {\vec {\dot{c}}}_\textrm{o} = ({\vec {\dot{f}}} - {\vec {\dot{{\hat{g}}}}})\frac{1}{1 - K_\textrm{a}} \end{aligned}$$

Here $\dot{{\hat{g}}}$ can be determined by assuming that the gravity does not tilt substantially away from the vertical. Such an assumption allows for the use of the small angle approximation. Here gravity is tilted about the x-axis due to the rotational velocity of the otolith angle error multiplied by the gain, $K_f$ Therefore the change in gravity is given as

$$\begin{aligned} {\vec {\dot{{\hat{g}}}}} = 0i, K_fc_{\textrm{oy}}j, -\omega _x b k \end{aligned}$$

As the rate of change in gravity $\dot{{\hat{g}}}$ is zero in all but the lateral perturbation direction this is the only relevant component of the magnitude conflict.Therefore, substituting the above equation

$$\begin{aligned} {\dot{c}_{\textrm{oy}}} = ({\dot{f}_y} - K_fc_{\textrm{oy}})\frac{1}{1 - K_\textrm{a}} \end{aligned}$$

Rearranging this gives

$$\begin{aligned} {\dot{c}_{\textrm{oy}}} + c_{\textrm{oy}}\frac{K_f}{1-K_\textrm{a}} = {\dot{f}_y}\frac{1}{1 - K_\textrm{a}} \end{aligned}$$

Taking the Laplace transform yields

$$\begin{aligned} \frac{c_{\textrm{oy}}}{f_y} = \frac{s\left( \frac{1}{1 - K_\textrm{a}}\right) }{s + \frac{K_f}{1 - K_\textrm{a}}} \end{aligned}$$
